# A scoping review of approaches used to develop plant-based diet quality indices

**DOI:** 10.1016/j.cdnut.2023.100061

**Published:** 2023-02-28

**Authors:** Laura E. Marchese, Sarah A. McNaughton, Gilly A. Hendrie, Kate Wingrove, Kacie M. Dickinson, Katherine M. Livingstone

**Affiliations:** 1 Institute for Physical Activity and Nutrition, School of Exercise and Nutrition Sciences, Deakin University, Geelong, Australia; 2 CSIRO Health and Biosecurity, Adelaide, Australia; 3 Caring Futures Institute, College of Nursing and Health Sciences, Flinders University, Adelaide, Australia

**Keywords:** plant-based diet, diet quality, scoping review, dietary assessment, index, validity, dietary patterns, a priori

## Abstract

Plant-based dietary patterns are comprised of a range of foods, and increasingly, diet quality indices are used to assess them and their associations with health outcomes. As the design of these indices varies, a review of existing indices is necessary to identify common features, strengths, and considerations. This scoping review aimed to synthesize the literature on plant-based diet quality indices by examining their *1*) basis for development, *2*) scoring methodology, and *3*) validation approaches. MEDLINE, CINAHL, and Global Health databases were systematically searched from 1980 to 2022. Observational studies were included if they assessed plant-based diets in adults, using an *a priori* methodology with food-based components. Studies conducted among pregnant/lactating people were excluded. Thirty-five unique plant-based diet quality indices were identified in 137 included articles published between 2007 and 2022. Indices were developed to reflect epidemiological evidence for associations between foods and health outcomes (*n* = 16 indices), previous diet quality indices (*n* = 16), country-specific dietary guidelines (*n* = 9), or foods from traditional dietary patterns (*n* = 6). Indices included 4 to 33 food groups, with fruits (*n* = 32), vegetables (*n* = 32), and grains (*n* = 30) the most common. Index scoring comprised of population-specific percentile cutoffs (*n* = 18) and normative cutoffs (*n* = 13). Twenty indices differentiated between healthy and less healthy plant-based foods when scoring intakes. Validation methods included construct validity (*n* = 26), reliability (*n* = 20), and criterion validity (*n* = 5). This review highlights that most plant-based diet quality indices were derived from epidemiological research, the majority of indices differentially scored healthy and unhealthy plant and animal foods, and indices were most often evaluated for construct validity and reliability. To ensure best practice use and reporting of plant-based dietary patterns, researchers should consider the basis for development, methodology, and validation when identifying appropriate plant-based diet quality indices for use in research contexts.

## Introduction

The global sustainable development goals of optimizing human and environmental health have accelerated interest in plant-based diets recently [1,2]. Plant-based diets have been associated with a reduced risk of a wide range of noncommunicable diseases, including cardiovascular diseases, type 2 diabetes, and cancer [[3], [4], [5], [6]]. The FAO of the United Nations and the World Health Organisation recommend that a sustainable healthy diet is one that is predominantly plant-based, which “includes whole grains, legumes, nuts and an abundance and variety of fruits and vegetables” and “can include moderate amounts of eggs, dairy, poultry and fish; and small amounts of red meat” [7]. Thus, plant-based diets can be broadly defined as dietary patterns characterized by high intakes of foods of plant origin, such as fruits and vegetables, and lower intakes of foods of animal origin, such as red meat and dairy products [8,9].

Research to date has used a variety of ways to assess the continuum of inclusion of plant- and animal-based foods and the different combinations of these foods that constitutes a plant-based diet [8,10]. Traditionally many studies have investigated the healthiness of diet type classifications, such as a vegan diet, where there is complete elimination of animal products, or a vegetarian diet, where dairy and eggs may still be consumed [8]. This results in binary classifications of study populations based on whether participants consume animal foods [11]. However, the foods that constitute a plant-based dietary pattern may be better described along a continuum with varying degrees of inclusion and exclusion of animal foods, such as in a flexitarian diet [12]. Hence, more recently, an increasing number of studies have used diet quality indices to characterize and score a plant-based diet [[13], [14], [15]].

Diet quality indices assess the quality and variety of foods in the diet on the basis of prior knowledge, including dietary guidelines and cultural ways of eating, and create a composite score reflecting compliance with the prespecified criteria [16,17]. Several diet quality indices reflect a plant-based diet. For example, the Healthy Nordic Food Index has a strong plant-based focus and scores diets based on 6 components typically consumed as part of a traditional Nordic diet [18]. However, more recently, specific plant-based diet quality indices have been developed to capture the continuum of plant and animal foods consumed. For example, the provegetarian food pattern by Martínez-González et al. [14] positively scores 7 plant-based foods and negatively scores 5 animal-based foods. In addition to this, to acknowledge that not all plant-based foods are beneficial for health, Satija et al. [15] created a series of plant-based indices to differentiate between healthy plant-based foods such as whole grains, and less healthy plant-based foods such as sugar-sweetened beverages. As such, these indices reflect an overall plant-based diet (PDI), a healthful plant-based diet (hPDI), and an unhealthful plant-based diet (uPDI) [15].

With many new plant-based diet quality indices being developed, and increasing research examining their associations with health outcomes, it is imperative that these indices are reviewed. Therefore, this scoping review aimed to identify and critically evaluate diet quality indices used for assessing plant-based diets among adult populations, examining their basis for development, construction methodology, and validity. This synthesis will enable future studies to select the most suitable index for their research question and to inform this field of research going forward.

## Methods

A scoping review was most suitable in the context of this study as plant-based dietary pattern research is an emerging field, and a scoping review would provide a broader overview of the topic without requiring predefined specific questions or analysis of the quality of the studies [19]. This scoping review was conducted and reported according to the PRISMA extension for Scoping Reviews reporting (PRISMA-ScR) guidelines [20] (Supplemental Table 1).

### Eligibility criteria

Original peer-reviewed cohort, case control, and cross-sectional studies were included if they assessed a plant-based diet using an *a priori* methodology. Specifically, studies were included if *1*) they had a case control, cohort, or cross-sectional study design; *2*) participants were adults ≥18 y of age, free living, and noninstitutionalized people/populations; *3*) dietary intake was described using a food-based index that: *i*) was based on national guidelines or other public health recommendations, traditional dietary patterns, or published research on healthful dietary patterns, and, *ii*) quantified dietary intake as a numerical value; *4*) they included the term “plant-based diet” or a synonymous term (for example, vegetarian, paleolithic, etc.) when describing the index in the abstract, introduction, or methods section of the manuscript, or, the index components reflected a plant-based diet (for example, Nordic diet reflects pescatarian diet), or, that animal components were included in the index but negatively scored; *5*) they were published in English; and *6*) they were published from 1980 to the date of the final search.

Studies were excluded if they *1*) were not peer-reviewed, had review, commentary, editorial, conference proceeding, or thesis study designs or nonobservational designs; *2*) were conducted on animals, individuals <18 y of age, and pregnant and/or lactating people; *3*) used *a posteriori* or empirically derived diet indices or indices with nutrient or lifestyle components; *4*) used a Mediterranean diet score, Healthy Eating Index (HEI), Alternative HEI (AHEI), Dietary Inflammatory Index (DII), or Dietary Approaches to Stop Hypertension (DASH) indices; *5*) were not published in English; and *6*) were published before 1980.

This review focused on food-based indices, which are indices that use foods or food groups rather than nutrients as dietary components of the index. Focusing only on food-based indices rather than nutrient-based indices is consistent with the increasing body of literature on the benefits of whole food approaches, as reflected in national dietary guidelines and previous reviews [[21], [22], [23]]. Indices that included alcohol were included in the review if they assessed intake of alcoholic beverages. If an index included “sugar,” “added sugar,” or “added sweets” as a component but did not describe the foods included in this group, then the index was excluded as it was assumed these included sugars added to manufactured food and drinks.

For feasibility, indices related to the Mediterranean diet were excluded from this scoping review because of the large quantity of previous literature [[24], [25], [26]]. Additionally, articles using dietary indices related to the HEI [27], AHEI [28], DII [29], or DASH [30] diets were excluded at the title and abstract screening stage as they contain nutrient components within their calculations.

### Search strategy

A systematic search of the literature was conducted in MEDLINE Complete, CINAHL complete, and Global Health databases between 1980 to the date of the final search (22 August, 2022). The search was limited to studies published after 1980 as the first dietary pattern studies were published in the early 1980s [[31], [32], [33]]. The key search terms “diet∗, dietary, food or eating” were combined with “quality, index, indices, score∗, pattern∗” using a 2-word proximity operator, then combined with AND for “plant-based, vegan” and synonyms, and lastly combined with AND for “cross-sectional, cohort, case control, observational.” Search terms were combined using Boolean operators and searched in the title or abstract. Filters were used to limit the results to those published in English, from academic journals and conducted in adults ≥18 y of age (age restriction not applied in Global Health). In addition, the reference lists of the included studies were hand searched by 1 author (LEM) to identify any relevant studies that were not identified during the original search. Studies identified from the reference lists were only included if they reported on the original development of the index.

### Study screening

Independent screening of the studies was conducted in 2 stages using Covidence. First, titles and abstracts were screened by 2 independent reviewers (LEM screened all, second reviewer shared among KML, KMD, KW, GAH, and SAM) to determine eligibility against the inclusion and exclusion criteria. Conflicts were resolved by KW, KML, and LEM. Secondly, the full texts of remaining studies were screened in duplicate (LEM screened all, second reviewer shared among KML and KMD). Consensus for the studies to be included was reached through author discussions.

Plant-based diet quality indices were defined as unique if the article *1*) was published as an index development article; or *2*) did not reference another plant-based diet quality index; or *3*) combined 2 or more indices into 1 index, such as the plant-based diet index [13]. If the original publication of the index was not conducted in an observational setting, we included the first use of the index from an observational article that met our inclusion criteria. This was to ensure we only included indices suitable for use in adult populations. Indices identified as subscores or submetrics by the authors in their original publication were included as a note on the original index, rather than presented as a unique index. The PDI, hPDI, and uPDI scores were presented separately as unique indices as they are used individually across multiple studies [34,35].

### Data extraction

Data extraction templates were developed in Microsoft Excel (LEM) with input from all authors. Data extraction was conducted by one author (LEM) and 20% was verified for accuracy by a second author (KMD). The data extracted and summarized included study design, country, population age and sex, and exposure variables (dietary assessment tool, index name, basis for index development, food component information, and index calculation), and outcome variables (validation, evaluation of health-related outcome, and results) [21,36]. The basis for development was assessed on how the index was derived, such as country-specific dietary guidelines, cultural ways of eating, or epidemiological evidence for associations of food components with health outcomes. In relation to methodology, data were extracted on the index food group components, calculation of the individual food groups, and total score. For scoring methods using cutoffs to calculate food group intake, indices were grouped into normative and percentile cutoffs [37]. Normative cutoffs used values derived from the current literature of associations between diet and health, whereas percentile cutoffs were scored by ranking participants within the study population and indicated their value within a certain percentage group, such as median or tertile [37]. Finally, information on validity was extracted relating to 3 main categories: construct validity, criterion validity, and reliability. To identify if construct validity was assessed, the article needed to describe whether the index could either differentiate diet quality independent of energy intake (EI) or identify dietary intake that differs by sociodemographic characteristics [21,36,38]. To identify if criterion validity was assessed, the article needed to describe whether the plant-based diet quality index was compared with another validated diet quality index [36,38]. Lastly, reliability was identified through the description of whether a similar outcome was obtained when the measurement was repeated or by assessing the degree to which each of the food groups influenced the final score [21,38,39].

### Synthesis of results

A narrative synthesis was conducted to summarize, appraise, and compare the main findings from the included studies. The number and characteristics of unique plant-based indices identified were presented first to highlight the scope of indices available. Indices were then examined according to their basis for development, scoring methodology, and validation to provide an overview of the 3 key criteria researchers should consider when selecting an index. Studies that had applied these indices were then grouped on the basis of the plant-based diet quality index used, as per previous reviews [36]. This approach enabled a critical review of how and to what extent studies had adapted the index for use, which will provide insights for researchers on the parameters to consider when adapting plant-based diet quality indices.

## Results

**FIGURE 1:**
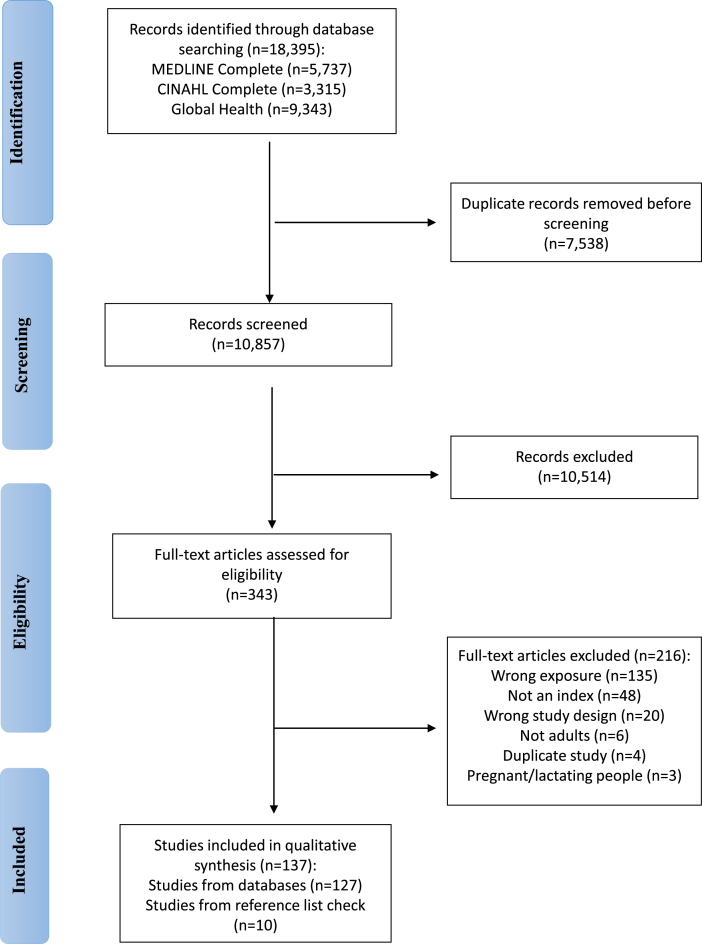
PRISMA flow diagram of search results from MEDLINE Complete, CINAHL Complete, and Global Health databases.

In total, 18,395 records were identified from a search of the 3 databases (Figure 1, PRISMA-ScR diagram). Duplicates were removed (*n* = 7538) resulting in 10,857 articles available for screening. After title and abstract screening, 10,514 articles were excluded. A total of 343 articles underwent full text screening, with 216 excluded, resulting in 127 studies eligible for inclusion and data extraction. A further 10 studies were identified from hand searching the reference lists of the final included studies. In total, 137 articles were included in this review.

### Study characteristics

Of the 137 included articles, 31 articles described the original development of 35 plant-based diet quality indices (Table 1 [[13], [14], [15],^18^,[40], [41], [42], [43], [44], [45], [46], [47], [48], [49], [50], [51], [52], [53], [54], [55], [56], [57], [58], [59], [60], [61], [62], [63], [64], [65], [66], [67], [68]] and Table 2 [15,18,[40], [41], [42], [43],[45], [46], [47], [48], [49],[51], [52], [53],^55^]), and the remaining 106 articles applied the 35 plant-based diet quality indices in an independent sample to the original development population (Supplemental Table 2). The 137 included articles were published from 2007 to 2022, with most published in 2022 (*n* = 39), 2021 (*n* = 41), 2020 (*n* = 20), and 2019 (*n* = 11).

**TABLE 1:** Overview of the development and methodology of the 35 plant-based diet quality indices

Index name	Reference and country	Development		Methodology				
		Basis for development	Dietary assessment tool	Number of food groups	Food groups	Calculation	Range	Score differentiated for healthy vs. less healthy plant-based foods
American Cancer Society diet score	McCullough et al. 2022 US [40]	Dietary recommendations from the 2020 American Cancer Society’s Guideline on Diet and Physical Activity for Cancer Prevention	FFQ	4	Fruit and vegetables, whole grains, red and processed meat, SSBs, and highly processed foods/refined grains	All groups scored from 0–3. Sex-specific consumption quartiles used for scoring all food groups (except for SSB). Fruit and vegetables group composed of 4 subgroups: vegetable intake, vegetable variety, fruit intake, fruit variety. Greater consumption of fruits and vegetables and whole grains scored higher points. SSBs and highly processed foods/refined grains composed of 2 subgroups: SSB intake, and highly processed/refined grains intake. SSBs scored using cutoff points for both sexes. Consumption of red and processed meat and SSBs and highly processed foods/refined grains was reverse scored. EI adjusted in analysis models.	0–12	Y, on the basis of cancer prevention guidelines
Animal-based diet quality index (aDQI)	Keaver et al. 2021 US [41]	*1*) PDI [15] *2*) Healthy Eating Index 2015 *3*) Alternative Healthy Eating Index *4*) American Heart Association score *5*) Epidemiological evidence for animal foods with health outcomes	24-h diet recall	6	Healthy animal group: fish/seafood, dairy, and poultry Unhealthful animal group: processed meats, red meats, and egg	Dietary intake of each food component was adjusted for total EI using density method, divided into sex-specific quintile cut offs, and each quintile scored from 0 to 5. Intake of each food group adjusted for total EI using density method. EI adjusted in analysis models.	0–30	N
*apriori* diet quality score	Meyer et al. 2013 US [42]	*1*) Hypothesized healthy foods *2*) Previously published indices [43,44]	Diet history	33	Beneficial: avocado, beans, beer, coffee, fish, fruit, green vegetables, lean fish, low-fat dairy, liquor, oil, other vegetable, poultry, seeds/nuts, soy products, tea, tomato, whole grains, wine, and yellow vegetables. Adverse: butter, fried foods, fried potatoes, grain dessert, organ meat, processed meat, regular red meat, salty snacks, sauces, soft drinks, sweet breads, sweet extra, and whole fat dairy	Food groups categorized into quintiles of consumption. Scored as 0 to 4 for beneficial or 4 to 0 for adverse food groups depending on study participants’ quintile level of consumption and summed across all 33 food groups. Food groups with a large proportion of nonconsumers, nonconsumers coded as 0 and consumers were categorized into quartiles. EI adjusted in analysis models.	0–132	Y, on the basis of epidemiological evidence for hypothesized health effects
*apriori* healthy diet pattern score	Lockheart et al. 2007 Norway [43]	*1*) Epidemiological evidence for food groups with myocardial infarction risk *2*) Principles of prudent v. Western diets from previous research	FFQ	28	Related to reduced MI risk: cheese and yogurt, low-fat dairy, tea, wine, beer, liquor, fruit, wholegrain breads, wholegrain breakfast cereals, low-fat fish, high-fat fish, chicken, nonhydrogenated vegetable oil, dressings, mayonnaise, and sauces, cruciferous vegetables, tomatoes, salad, other vegetables, nuts, and soup. Contribute to MI risk: high-fat milk, butter and margarine, high-energy drinks, liver, red and processed meats, chips and snacks, sweets, and pizza	Sum of tertile rankings - tertiles of intake, using g/d cutoff points calculated from the control group, assigned values 0 for the lowest, 1 for the middle and 2 for the highest for the groups related inversely to MI. Scoring reversed for the food groups that contribute to MI risk. EI adjusted in analysis models.	0–56	Y, on the basis of epidemiological evidence indicating a positive or inverse association between the plant food and health outcomes
Comprehensive Diet Quality Index (cDQI)	Keaver et al. 2021 US [41]	*1*) PDI (15) *2*) Healthy Eating Index 2015 *3*) Alternative Healthy Eating Index *4*) American Heart Association score *5*) Epidemiological evidence for animal foods with health outcomes	2 × 24-h diet recalls	17	Healthy plant group: whole grains, vegetables (excluding white potatoes), whole fruits, nuts/seeds/legumes, vegetable oils, and coffee/tea Unhealthful plant group: refined grains, fruit juices, white potatoes, SSBs, and sweets/desserts Healthy animal group: fish/seafood, dairy, poultry Unhealthful animal group: processed meats, red meats, and egg	Dietary intake of each food component was adjusted for total EI using density method, divided into sex-specific quintile cut offs, and each quintile scored from 0 to 5. Healthy animal foods and healthy plant foods positively scored, and unhealthy animal foods, and unhealthy plant foods reverse scored. EI adjusted in analysis models.	0–85	Y, on the basis of epidemiological evidence indicating a positive or inverse association between the plant food and health outcomes
Dietary Obesity-Prevention Score (DOS)	Gómez-Donozo et al. 2019 Spain [45]	Epidemiological evidence for foods associated with weight changes	Semiquantitative FFQ	14	Vegetables, fruits, legumes, yogurt, nuts, fish, and vegetable-to-animal protein ratio, red meat, processed meat, saturated animal fat, refined grains, ultraprocessed foods, SSBs, beer, and spirits	Energy adjusted intakes (g/d) ranked into sex-specific tertiles. Tertiles scored 1–3. Positively weighted the consumption of vegetables, fruits, legumes, yogurt, nuts, fish, and vegetable-to-animal protein ratio; consumption of red meat, processed meat, saturated animal fat, refined grains, ultraprocessed foods, SSBs, beer and spirits were inversely weighted. EI adjusted in some analysis models.	14–42	Y, on the basis of epidemiological evidence indicating a positive or inverse association between the plant food and health outcomes
Dietary phytochemical index	Vincent et al. 2010 US [46]	Constructed on the basis of published phytochemical index	3-d dietary record	10	Fruits and vegetables (and prepared foods derived from these), legumes, whole grains, seeds, nuts, fruit or vegetable juices, olive oil, soy sources, wine, and beer and cider	Calculated as the percentage of the daily energy derived from phytochemical-rich foods divided by the total daily caloric intake = (phytochemical kJ/total kJ) × 100.	0–100	N
Dietary quality score	van der Velde et al. 2022 Netherlands [47]	Dutch Dietary Guidelines (DDG)	FFQ	13	Vegetables, fruit, legumes, unsalted nuts, fish, grain products, dairy, tea, coffee, oils and fats, SSBs, savory snacks, and sweet snacks	Each food group scored from 0–10. Vegetables (g/d), fruit (pieces/d), legumes (g/wk) and unsalted nuts (g/d) scored continuously with higher intake scoring higher points. Fish divided into 2 subgroups on the basis of serves per week and fat type. Grain products divided into 2 subgroups on the basis of g/d and grain type. Dairy divided into 2 subgroups on the basis of served per day and fat type. Tea divided into 2 subgroups on the basis of s/d and type. Coffee scored on the basis of type. Oils and fats divided into 2 subgroups on the basis of fat type for cooking, and fat used on bread. SSBs, savory snacks and sweet snacks scored continuously with higher consumption resulting in lower scores.	0–130	Y, on the basis of recommendations from the DDG
Diet Quality Index Associated to the Digital Food Guide (DQI-DFG)	Caivano et al. 2019 Brazil [48]	*1*) Guidelines of the School of Public Health at Harvard University *2*) Brazilian food habits	24-h recall	11	Moderation components: sugars and sweets, meats: beef, pork and processed meat, refined cereals, and processed fats Adequacy components: poultry, fish and eggs, whole cereals, tubers and roots, fruits, vegetables, legumes and oilseeds, milk and dairy products, and oils and fats	All food groups scored a maximum of 5 or 10, on the basis of portion per 1,000 kcal. Dichotomous scoring for moderation components, where the maximum score was awarded for being within the recommended intake range or scored 0 when this range exceeded. For adequacy components, increasing score for higher intake with the maximum score awarded for being within or above the recommended intake range. For whole cereals, tubers and roots, fruits, and milk and dairy products with intake above the recommendation, decreasing scores were given for intake that is twice the upper limit, and no points for intake above twice the upper limit.	0–100	Y, on the basis of evidence between foods and health, and meeting nutritional recommendations
Diet Quality Score	Toft et al. 2007 Denmark [49]	*1*) Indices of overall diet quality [50] *2*) The Danish Dietary Guidelines	FFQ	4	Vegetables (cooked or raw) and/or vegetarian dishes, fruit, fish, and fat	Each food group scored from 1–3 points on the basis of defined frequency of consumption groups. Vegetables scored on servings per week, fruit scored by pieces per day, and fish scores on grams per week. Fat scored on the basis of fat type applied to bread and cooking.	1–12	N
Diet Score	Jannasch et al. 2022 Germany [51]	*1*) German dietary guidelines *2*) Epidemiological evidence for food groups with risk of chronic diseases	FFQ	10	Bread and cereals overall intake, fermented dairy products, raw and cooked vegetables, fruits, legumes, unsalted nuts, fish overall intake, meat and processed meat, vegetable oils intake, and SSBs	All food groups scored from 0 to 1 on the basis of portions per day or week. Bread and cereals overall intake, fish overall intake, and meat and processed meat, divided into 2 subgroups on the basis of portions and type, scored 0 to 0.5 each. Vegetable oils intake divided into 2 subgroups on the basis of intake frequency and general use and scored 0 to 0.5 each. Higher scores attained for lower meat consumption.	0–10	Y, on the basis of epidemiological evidence indicating a positive or inverse association between the plant food and health outcomes, as well as German dietary guidelines
Dutch Dietary Guidelines (DDG) Index	Moazzen et al. 2022 Netherlands [52]	*1*) Epidemiological evidence for the impact of food components and dietary habits in the development of diet-related chronic diseases *2*) DDG *3*) Netherlands Nutrition Centre surveys	FFQ	13	Vegetables, fruits, legumes, whole grain products, unsalted nuts, fish, soft margarine, liquid cooking fats, vegetable oil, tea, red and processed meat, SSBs, and alcohol	All food groups have a cutoff score for sufficient consumption. Intake scored as adhering (1) or not adhering (0) to the recommended amount per food group. EI adjusted in analysis models.	0–13	Y, on the basis of epidemiological evidence indicating a positive or inverse association between the plant food and health outcomes, as well as the DDG
Global Diet Quality Score (GDQS)	Bromage et al. 2021 African countries, China, India, Mexico, US [53]	*1*) Prime diet quality score [54] *2*) Nutritionally important foods in low-to-middle income countries *3*) Epidemiological evidence: associations between foods and health	FFQ and 24-h recall	25 Submetrics GDQS+: 16 GDQS−: 9	Healthy food groups: citrus fruits, deep orange fruits, other fruits, dark green leafy vegetables, cruciferous vegetables, deep orange vegetables, other vegetables, deep orange tubers, legumes, nuts and seeds, whole grains, liquid oils, fish and shellfish, poultry and game meat, low-fat dairy, and eggs Unhealthy groups when consumed in excess: high-fat dairy and red meat Unhealthy food groups: processed meat, refined grains and baked goods, sweets and ice cream, SSBs, juice, white roots and tubers, and purchased deep fried foods	All foods ranked into 3 categories of consumed amounts (g/d) (apart from high-fat dairy which has 4 categories). The categories of consumption (g/d) are specific for each food group. Points are assigned on the basis of these categories. Healthy foods have higher scores for higher consumption. Unhealthy when consumed in excess foods are scored in increasing points until specific amounts are consumed. Unhealthy foods are given more points for lower consumption. All groups scored from 0 to 2 points, apart from: cruciferous vegetables, deep orange vegetables, other vegetables, and deep orange tubers (0–0.5 points); red meat (0–1 point); dark green leafy vegetables, legumes, nuts and seeds (4 points). EI adjusted in some analysis models. Submetrics: GDQS+: same scoring as GDQS, but only includes the healthy food groups GDQS-: same scoring as GDQS, but only includes the 9 unhealthy food groups	0–49 Submetrics: GDQS+: 0–32 GDQS-: 0–17	Y, on the basis of epidemiological evidence indicating a positive or inverse association between the plant food and health outcomes
Healthful plant-based diet index (hPDI)	Satija et al. 2016 US (15)	*1*) Provegetarian food pattern (14) *2*) Epidemiological evidence for associations with health outcomes	Semiquantitative FFQ	18	Healthy plant group: whole grains, fruits, vegetables, nuts, legumes, vegetable oil, and tea and coffee. Less healthy plant group: fruit juices, refined grains, potatoes, SSBs, sweets, and desserts. Animal group: animal fat, dairy, eggs, fish and seafood, poultry, unprocessed red meat, processed red meat, and miscellaneous animal-based food	Each component divided into quintiles on the basis of servings per day, and each quintile scored from 1 to 5. Healthy plant foods positively scored, less healthy plant foods and animal foods negatively scored. EI adjusted in analysis models.	18–90	Y, on the basis of epidemiological evidence indicating a positive or inverse association between the plant food and health outcomes
Healthy Eating Quiz (HEQ) score	Williams et al. 2017 Australia [55]	Australian Recommended Food Score (ARFS)	HEQ	7	Vegetables, fruit, vegetarian sources of protein (nuts, nut butters, eggs, soybeans or tofu, baked beans, other beans or lentils), breads and cereals, dairy, water, and spreads/sauces	Score for vegetarians: food groups comprised of individual food items which are awarded one point for a consumption frequency of at least once per week (some awarded 2 points). Double points for vegetarian questions with ≥1 /wk consumption, and one bonus point if both soybeans, tofu and other beans, lentils were consumed ≥1 /wk. All points summed for total score.	0–73	N
Healthy Nordic Food Index	Olsen et al. 2011 Netherlands [18]	*1*) Traditional Nordic food items *2*) Epidemiological evidence with beneficial health outcomes	FFQ	6	Fish, cabbages, whole grain rye (eaten as rye bread), whole grain oats (eaten as oatmeal), apples and pears, and root vegetables.	Fish, cabbages, apples and pears, root vegetables - 1 point given for intake above the sex-specific median. Whole grain rye bread and whole grain oatmeal - scorings based on sex-specific spline curves with boundaries at the predefined questionnaire categories. Cutoff values defined by the boundary indicating the largest alteration in the path of the curve, and one point was given for: rye bread ≥2 slices/d (63 g/d) and oatmeal ≥2 portions/wk (21 g/d). EI adjusted in some analysis models.	0–6	N
HEI-flexible (HEI-flex)	Bruns et al. 2022 Germany [56]	*1*) HEI-2015 (39) *2*) Food intake data from the FFQ *3*) Questions regarding plant-based alternatives *4*) Recommendations of the German Nutrition Society guidelines *5*) WHO guidelines *6*) Dietary guidelines for Americans	FFQ, plant-based alternative products questionnaire, 3-d diet diary	14	Beverages, vegetables, fruit, protein sources, carbohydrate sources, whole meal, nuts and seeds, processed meat and plant-based meat alternatives, milk and dairy products and plant-based dairy alternatives, alcohol, high-energy density foods (sweet), high-energy density foods (fat), drinks with high-energy density, and fats and oils and plant-based fat substitutes	Mean daily intake was calculated for each food group by a formula. Ratio between intake and intake recommendation calculated for each food group to create a unique HEI-flex score on the basis of adequacy and moderation principles. Food groups could score a maximum of 100 points, which was divided by 14 to create the final total.	0–100	Y, on the basis of official consumption recommendations from dietary guidelines and WHO
Japanese Diet Index Score	Tomata et al. 2014 Japan [57]	*1*) Dietary pattern derived by factor analysis *2*) Reported foods part of traditional Japanese diet	FFQ	9	Positive: rice, miso soup, seaweeds, pickles, green and yellow vegetables (green vegetables, carrot, pumpkin, tomato), fish (raw fish, fish boiled with soy, roast fish, boiled fish paste, dried fish), and green tea Negative: beef and pork (beef, pork, ham, and sausage) and coffee	Positive component: 1 point if intake was more than or equal to the sex-specific median. Negative components: 1 point if intake was below the sex-specific median. EI adjusted in some analysis models.	0–9	N
Japanese Food Guide Spinning Top	Oba et al. 2009 Japan [58]	Dietary Guidelines for Japanese	Semiquantitative FFQ	7	Grain dishes (including rice, bread, and noodles), vegetable dishes (including vegetables, mushrooms, potatoes, and seaweed), fish and meat dishes (including meat, fish, eggs, and soybeans), milk (milk and milk products), and fruits (fruits and 100% fruit juice counted as half the weight), energy from total diet and energy from snacks or alcoholic beverages	The recommended number of servings by food group and the recommended total EI specified according to sex, age, and 2 levels of physical activity. Consuming the recommended number of servings (or energy) received a score of 10 for that group. Exceeding or falling short of the recommended servings or energy - the score was calculated proportionately between 0 and 10. EI adjusted in analysis models.	0–70	Y, on the basis of Dietary Guidelines for Japanese
Japanese food score	Okada et al. 2018 Japan [59]	Previous studies reporting dietary patterns of the Japanese diet from principal component/factor analysis	FFQ	7	Beans and beans products (boiled beans and tofu), fresh fish, vegetables (spinach or garland chrysanthemum, carrots or pumpkin, tomatoes, cabbage or head lettuce, and Chinese cabbage), Japanese pickles, fungi, seaweeds, and fruits (citrus fruit and others)	Food component intake ≥3–4 times/wk was considered as the cutoff point (1 point is given) in participants who ate any food item from the 7 food groups. EI adjusted in some analysis models.	0–7	N
Lifelines Diet Score (LLDS)	Vinke et al. 2018 Netherlands [60]	*1*) 2015 DDG *2*) Epidemiological evidence for diet disease relationships at the food group level	Semiquantitative FFQ	12	Positive groups: vegetables, fruit, whole grain products, legumes and nuts, fish, oils and soft margarines, unsweetened dairy, and coffee and tea Negative groups: red and processed meat, butter and hard margarines, and SSBs	Intake of the food groups calculated in grams per 1000 kcal. For each food group, intake divided into population-specific quintiles. Four points awarded for the highest consumption of positive food groups, and to the lowest quintile for negative food groups.	0–48	Y, on the basis of DDG and on epidemiological evidence indicating a positive or inverse association between the plant food and health outcomes
Nordic diet score	Galbete et al. 2018 Germany [61]	*1*) Previous publications about the Nordic diet *2*) FFQ data	Semiquantitative FFQ	9	Whole grain and rye bread, berries, apples and pears, fish, cabbage and cruciferous vegetables, root vegetables, low-fat dairy products, potatoes, and vegetable fats (excluding olive oil)	Each food component categorized into sex-specific tertiles of intake and scored 0–2 points according to the first, second, and third tertile. EI adjusted in some analysis models.	0–18	N
Plant-based dietary diversity score (DDS)	Liu et al. 2021 China [62]	Published DDS [63]	FFQ	6	Fresh vegetables, preserved vegetables, fresh fruit, tea, garlic, and food made from beans	Consumption frequency of all food groups. Scored 0 (rarely), 1 (occasionally), and 2 (often) without a minimum intake.	0–12	N
Plant-based diet index	Chen et al. 2018 Netherlands [13]	*1*) Dutch diet *2*) DDG *3*) Provegetarian food pattern (14) *4*) PDI (15)	Semiquantitative FFQ	23	Plant group: fruit, vegetables, whole grains, nuts, legumes, potatoes, vegetable oils, tea and coffee, SSB, refined grains, sweets, and alcoholic beverages Animal group: low-fat milk, full-fat milk, low-fat yogurt, full-fat yogurt, cheese, unprocessed lean meat, fish, eggs, animal fat, desserts/dairy with sugars, and processed meat/red meat	Grams per day for each component divided into quintiles on the basis of cohort intake, and each quintile scored from 0 to 4. Plant components positively scored; animal foods reverse scored. EI adjusted in analysis models.	0–92	N
Plant-based diet index (PDI)	Satija et al. 2016 US [15]	*1*) Provegetarian food pattern [14] *2*) Epidemiological evidence for associations with health outcomes	Semiquantitative FFQ	18	Healthy plant group: whole grains, fruits, vegetables, nuts, legumes, vegetable oil, tea & coffee Less healthy plant group: fruit juices, refined grains, potatoes, SSBs, sweets and desserts Animal group: animal fat, dairy, eggs, fish and seafood, poultry, unprocessed red meat, processed red meat, and miscellaneous animal-based food	Each component divided into quintiles on the basis of servings per day, and each quintile scored from 1 to 5. Plant foods positively scored, animal foods negatively scored. EI adjusted in analysis models.	18–90	Y, on the basis of epidemiological evidence indicating a positive or inverse association between the plant food and health outcomes
Plant-based diet index	Yang et al. 2021 China [64]	FFQ data	FFQ	12	Plant food group: staple food, fruits, vegetables, beans, nuts, cereal, pickles Animal food group: livestock, poultry, fish, egg, and dairy	Food groups ranked into quartiles on the basis of average daily intake in g/d. All food groups scored from 1–4. Plant foods positively scored; animal foods reverse scored. EI adjusted in some analysis models.	12–48	N
Plant-based Diet Quality Index (pDQI)	Keaver et al. 2021 US [41]	*1*) PDI [15] *2*) Healthy Eating Index 2015 *3*) Alternative Healthy Eating Index *4*) American Heart Association score *5*) Epidemiological evidence for animal foods with health outcomes	24-h diet recall	11	Healthy plant group: whole grains, vegetables (excluding white potatoes), whole fruits, nuts/seeds/legumes, vegetable oils, and coffee/tea Unhealthful plant group: refined grains, fruit juices, white potatoes, SSBs, and sweets/desserts	Dietary intake of each food component was adjusted for total EI using density method, divided into sex-specific quintile cut offs, and each quintile scored from 0 to 5. Healthy plant foods positively scored, and unhealthy plant foods reverse scored. EI adjusted in analysis models.	0–55	Y, on the basis of epidemiological evidence indicating a positive or inverse association between the plant food and health outcomes
Plant-based food variety score (PFVS)	Zhou et al. 2020 China [65]	Based on a general food variety score	FFQ	5	Whole grain cereals, legumes, vegetables, fruits, and nuts	One point was awarded for each food item consumed at least twice a week. The food items and their corresponding points for the total FVS are: whole grain cereals (1), legumes (4), vegetables (22), fruits (9), and nuts (4).	0–40	N
Plant diet	Purnamasari et al. 2022 Taiwan [66]	Not outlined	FFQ	8	Rice and flour products, whole grains, rhizomes, bread, light-colored vegetables, dark-colored vegetables, beans and legumes, and fruits	The score for each food group was assigned from 1 to 5, from the lowest to the highest intake frequency. Median plant score from study participants used to define high (≥median) or low (<median) intake.	8–40	N
Plant food score (PFS)	Dennis et al. 2021 US [67]	Major dietary factors contributing to consumption of metal-binding plant compounds	FFQ	7	Plant foods: fruit, vegetable, legumes, nuts/seeds, and whole grains Beverages: tea and wine	All plant foods adjusted for total EI using grams/kcal. Foods - categorized into low, medium, and high intake categories by sex-specific tertiles. Low through high categories were assigned 0–2 points. Beverages–consumption of ≥1 cup of tea/d or 1 drink of wine/wk received 1 point, everyone else received 0 points. EI adjusted in analysis models.	0–12	N
Prime Diet Quality Score (PDQS)	Fung et al. 2018 US [54]	*1*) Prime Screen questionnaire *2*) Epidemiological evidence between foods with risk of noncommunicable diseases *3*) Nutrient contribution in the worldwide setting	FFQ	21	Healthy food groups: dark leafy green vegetables, cruciferous vegetables, carrots, other vegetables, whole citrus fruits, other whole fruits, legumes, nuts, poultry, fish, eggs, whole grains, and liquid vegetable oils Unhealthy food groups: red meat, potatoes, processed meat, whole milk dairy, refined grains and baked goods, SSB, fried foods obtained away from home, and desserts and ice cream	Scoring criteria: 0–1 serving/wk (0 point), 2–3 servings/wk (1 point), ≥4 servings/wk (2 points) for the healthy food groups. Scoring was reversed and points deducted for the unhealthy food groups. Points for each food group were summed to create the final score. EI adjusted in analysis models.	0–42	Y, on the basis of epidemiological evidence indicating a positive or inverse association between the plant food and health outcomes
Provegetarian food pattern	Martínez-González et al. 2014 Spain [14]	Epidemiological evidence for associations between dietary patterns and mortality	Semiquantitative FFQ	12	Plant group: fruit, vegetables, nuts, cereals, legumes, olive oil, and potatoes. Animal group: added animal fats, eggs, fish, dairy products, and meats and meat products	Energy adjusted grams per day divided into sex-specific quintiles for each component, and each quintile scored from 1 to 5. Plant groups positively scored; animal groups negatively scored. EI adjusted in some analysis models.	12–60	N
Simplified Healthy Dietary Pattern	Nettleton et al. 2008 US [44]	Epidemiological evidence: associations between food groups and CVD outcomes	FFQ	6	Whole grain bread, rice, cereal or pasta, fruit, and nuts/seeds, added fats and oils, processed meats, and fried potatoes	Whole grains, fruit, and nuts/seeds ranked by servings per day (4 categorical ranks multiplied by +1), added fats and oils, processed meats, and fried potatoes (4 categorical ranks multiplied by −1). EI adjusted in analysis models.	−9 to +9	Y, on the basis of epidemiological evidence indicating a positive or inverse association between the plant food and health outcomes
TOT-Diet score	van der Velde et al. 2020 Netherlands [68]	*1*) Food intake from the DDG *2*) Food choices from the Health Council of the Netherlands and the Netherlands Nutrition Centre	FFQ	6	Vegetables, fruit, fish, bread, oils and fats, and sweet and savory snacks	Each food group scored 0–10. Fruits (pieces/d) and vegetables (g/d) received higher scores for higher consumption. The score for fish comprised of 2 subgroups: number of servings per wk, and the fat quality of the fish. Bread was divided into 2 subgroups: type of bread, and sex-specific intakes of wholegrain sandwiches per day. Fats and oils were divided into 2 subgroups: the type of fat for cooking, the type of fat for bread. Sweet and savory snacks were divided into 4 subgroups, where higher consumption received lower scores.	0–60	Y, on the basis of DDG and food intake in the Netherlands
Unhealthful plant-based diet index (uPDI)	Satija et al. 2016 US [15]	*1*) Provegetarian food pattern [14] *2*) Epidemiological evidence for associations with health outcomes	Semiquantitative FFQ	18	Healthy plant group: whole grains, fruits, vegetables, nuts, legumes, vegetable oil, and tea and coffee Less healthy plant group: fruit juices, refined grains, potatoes, SSBs, sweets, and desserts Animal group: animal fat, dairy, eggs, fish and seafood, poultry, unprocessed red meat, processed red meat, and miscellaneous animal-based food	Each component divided into quintiles on the basis of servings/d, and each quintile scored from 1 to 5. Less healthy plant foods positively scored, healthy plant foods and animal foods negatively scored. EI adjusted in analysis models.	18–90	Y, on the basis of epidemiological evidence indicating a positive or inverse association between the plant food and health outcomes

N, no; DDS, dietary diversity score; DDG, Dutch Dietary Guidelines; FFQ, food frequency questionnaire; PDI, plant-based diet index; SSB, sugar-sweetened beverage; Y, yes.

**TABLE 2:** Overview of the validity of the 35 plant-based diet quality indices

General		Population			Construct validity			Criterion validity		Reliability			Article objective(s) included index validity		Outcome(s) assessed
Index name	Observational study design	Studied population age	Population size	Sex	Associated with selected nutrients in expected directions^1^		Score differences between groups with established differences in diet	Index correlated with another validated *a priori* diet quality index		Similar outcome obtained when the measurement was repeated		Assessed the degree to which each of the food groups influences the final score			
American Cancer Society diet score [40]	PCS	≥30 y	155,331	79% F			A						N		Socio economic and geographic factor associations
Animal-based diet quality index (aDQI) [41]	PCS	≥20 y	36,825	51% F			A						N		Mortality
*apriori* diet quality score [42]	LCS	≥18 y	2718	M & F (% not reported)	A		A			A			N		F2-isoprostanes
*apriori* healthy diet pattern score [43]	CC	≥45–75 y	211	M & F (% not reported)									N		MI
Comprehensive Diet Quality Index (cDQI) [41]	PCS	≥20 y	36,825	51% F			A					A	N		Mortality
The Dietary Obesity-Prevention Score (DOS) [45]	PCS	35 ± 11 y at baseline	11,349	73% F	A		A			A		A	N		Overweight/obesity and average yearly weight changes
Dietary phytochemical index [46]	CS	18–30 y	54	65% F			A						N		Annual weight gain, adiposity, oxidative stress and inflammation
Dietary quality score [47]	CS	18–88 y	1033	48% F			A			A			N		Theory of planned behavior
Diet Quality Index Associated to the Digital Food Guide (DQI-DFG) [48]	CS	19–60 y	664	M & F (% not reported)						A		A	Y		Improve and validate the index
Diet Quality Score [49]	CS	30–60 y	6542	M & F (% not reported)	A		A						Y		Develop the index and CVD risk factors
Diet Score [51]	PCS	35–67 y	134	44% F			A			A		A	Y		Dietary intake
The Dutch Dietary Guidelines (DDG) Index [52]	PCS	≥40 y	72,695	57% F									N		Gastrointestinal cancer risk
Global Diet Quality Score (GDQS)^2^ [53]	C	≥25 y	65,288	100% F			A						Y		Nutrient adequacy and diet-related NCD risk
Healthful plant-based diet index (hPDI) [15]	PCS	≥25 y	200,727	80% F	A		A	A (aMED, AHEI, and DASH)		A			N		T2D
Healthy Eating Quiz (HEQ) score^3^ [55]	CS	≥25 y	4,623	M & F (% not reported)									N		Diet quality
Healthy Nordic Food Index [18]	PCS	≥50 y	50,290	54% F			A			A			N		Mortality
HEI-flexible (HEI-flex) [56]	CS	25–45 y	94	52% F	A							A	Y		Diet quality
Japanese Diet Index Score [57]	PCS	≥65 y	14,260	55.2% F									N		Incident functional disability
Japanese Food Guide Spinning Top [58]	PCS	≥35 y	29,079	54% F	I		A			A			N		Mortality
Japanese food score [59]	PCS	≥40 y	58,767	58% F	A		A						N		All-cause, CVD, and cancer mortality
Lifelines Diet Score (LLDS) [60]	PCS	≥18 y	129,369	58.5% F			A					A	N		Discriminative capacity of score and sociodemographic determinants
Nordic diet score [61]	PCS	≥35 y	23,485	61% F	A		A	A (Mediterranean diet scores)					N		T2D, MI, stroke, and cancer
Plant-based dietary diversity score [62]	PCS	≥65 y	17,959	56% F									N		All-cause mortality
Plant-based diet index (PDI) [13]	PCS	≥45 y	6798	59% F						A		A	N		Insulin resistance, prediabetes, and T2D
Plant-based diet index (PDI) [15]	PCS	≥25 y	200,727	80% F	A		A	A (aMED, AHEI, and DASH)		A			N		Risk of T2D
Plant-based diet index [64]	C	18–79 y	37,985	61% F			A			A			N		Risk of T2D
Plant-based Diet Quality Index (pDQI) [41]	PCS	≥20 y	36,825	51% F			A						N		Mortality
Plant-based food variety score (PFVS) [65]	CS	20–45 y	248	100% F						A			N		Risk of uterine fibroids
Plant diet^4^ [66]	RCS	20–45 y	22,631	100% F			A			A			N		Anemia
Plant food score (PFS) [67]	PCS	≥45 y	1901	51% F	A		A			I			N		Urinary creatinine- adjusted cadmium
Prime Diet Quality Score (PDQS) [54]	PCS	≥27 y	212,142	79% F	A		A			A			N		Ischemic heart disease
Provegetarian food pattern [14]	PCS	≥55 y	7216	57% F	A		A	I (Mediterranean diet score)		A			N		All-cause mortality
Simplified Healthy Dietary Pattern [44]	CS	≥45 y	5089	53% F			A						N		Markers of CVD risk
TOT-Diet score [68]	CS	≥18 y	242	M & F (% not reported)			A						N		Food insecurity, obesity, and sociodemographic and lifestyle factors
Unhealthful PDI (uPDI) [15]	PCS	≥25y	200,727	80% F				A (aMED, AHEI, and DASH)		A			N		T2D
Totals					A	I	A	A	I	A	I	A		Y	N
					(*n* = 11)	(*n* = 1)	(*n* = 25)	(*n* = 4)	(*n* = 1)	(*n* = 16)	(*n* = 1)	(*n* = 7)		(*n* = 5) 14%	(*n* = 30)86%

A, adequate association; AHEI, Alternative Healthy Eating Index; aMED, alternative Mediterranean Diet; C, cohort; CC, case control; CS, cross-sectional, CVD, cardiovascular disease; F, female; I, inadequate association; LCS, longitudinal cohort study, M, male; N, no; NCD, noncommunicable disease; PCS, prospective cohort study, RCS, retrospective cohort study; T2D, type 2 diabetes; Y, yes. ^1^Independent of EI and in expected directions for good diet quality, as concluded by the article authors or identified by the authors of this review. ^2^Results only included from cohort datasets conducted on adults. ^3^Results reporting the HEQ score for vegetarians and for respondents ≥18 y. ^4^Results for combined high plant diet score and low animal diet score.

### Plant-based diet quality indices

**FIGURE 2:**
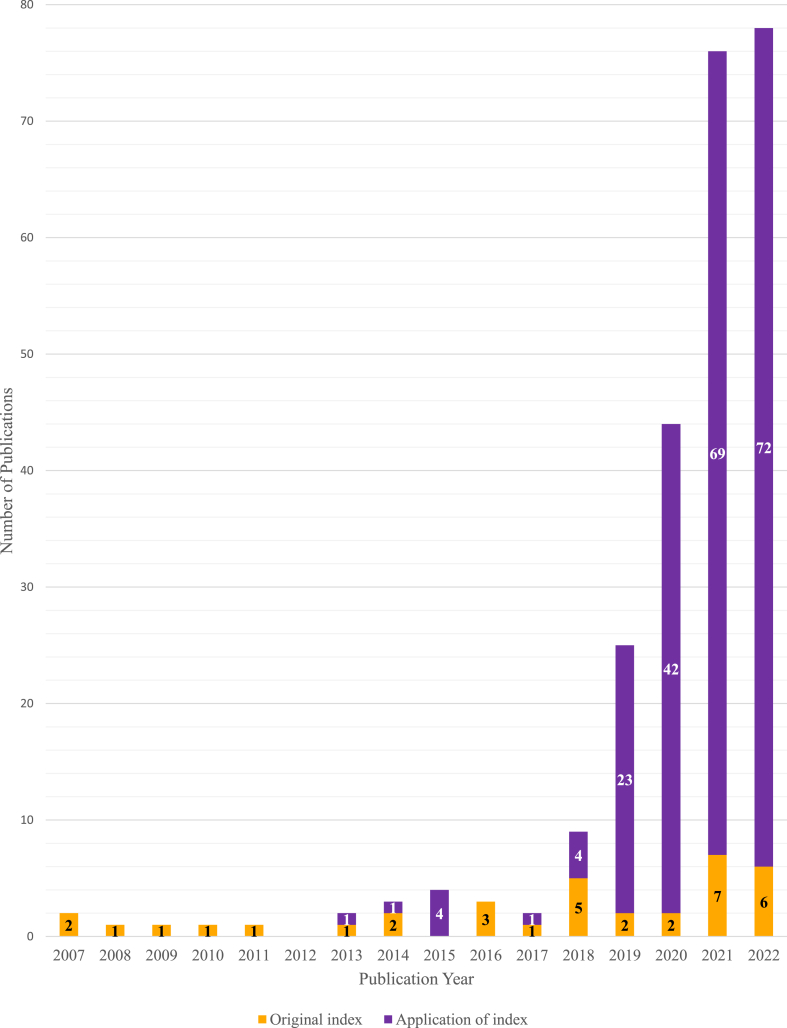
Number of original plant-based diet quality indices published, and the number of publications applying them per year.

Across the 137 articles, 35 unique plant-based diet quality indices were reported. The indices were initially published in 31 articles between 2007 (*n* = 2) [44,50] to 2022 (*n* = 6) [43,44,48,53,57,67], but most were first published after 2017 (*n* = 20) [13,41,42,46,48,49, [52], [53], [54], [55],^57^,[60], [61], [62], [63],[65], [66], [67], [68], [69]] (Figure 2 and Table 1). The indices were mostly developed using populations from the US (*n* = 13) [15,[41], [42], [43],^45^,^47^,^54^,^55^,^68^], followed by the Netherlands (*n* = 6) [13,18,48,53,61,69] and China (*n* = 2) [54,63,65,66]. The sample sizes of the development studies ranged from 54 [47] to 212,142 [55], with almost all conducted in samples of men and women (*n* = 32) [13,14,15,18, 41–50,52,53,55–63,65,68,69) (Table 2). About half of the indices (*n* = 21) were developed using data from prospective cohort studies [13,14,15,18,41,42,46,[51], [52], [53], [54], [55],^59^,^60^,^62^,^63^,^68^] followed by cross-sectional studies (*n* = 9) [45,[47], [48], [49], [50],^56^,^57^,^66^,^69^].

### Development of plant-based diet quality indices

The basis of development for each index was identified and categorized into 4 groups: *1*) previously published diet quality indices; *2*) epidemiological evidence for associations between food groups and health outcomes; *3*) country-specific dietary guidelines; and *4*) traditional dietary patterns. Almost half (46%, *n* = 16) of the indices used a previously published diet quality index as part of their basis for development [13,15,42,43,50,54,[56], [57], [58],^60^,^63^,^66^]. Of these, most plant-based indices were developed on the basis of well-established diet quality indices, such as the HEI [23], AHEI [24], or previously published plant-based diet quality indices, such as PDI [15] and the provegetarian food pattern [14]. For example, HEI-flex was developed on the basis of the HEI-2015 [39]. About 46% of indices (*n* = 16) used epidemiological evidence for associations between food groups and health outcomes as the basis for their development [14,15,42,[44], [45], [46],[52], [53], [54],^61^]. For example, when developing the provegetarian food pattern, Martínez-González et al. [14] used a prospective cohort study to examine evidence for associations between dietary patterns and mortality, whereas others have used evidence for associations with body weight [46], or risk of noncommunicable diseases [45,55]. Nine indices used country-specific dietary guidelines [13,48,50,52,53,57,59,61,69], and 6 used food groups from traditional dietary patterns [13,18,49,58,60,62]. The most used dietary guidelines were the Dutch Dietary Guidelines (*n* = 5) [13,53,48,61,69]. To develop the plant-based diet quality indices, dietary intake data was collected using a variety of dietary assessment tools. Most indices used a food frequency questionnaire (FFQ) as the dietary assessment tool (*n* = 19) [18,41,44,45,48,50,[52], [53], [54], [55],^57^,^58^,^60^,^63^,[65], [66], [67], [68], [69]], followed by a semiquantitative FFQ (*n* = 9) [[13], [14], [15],^46^,^59^,^61^,^62^] or 24-h dietary recall (*n* = 5) [42,49,54] (Table 1).

### Methodology of constructing plant-based diet quality indices

**FIGURE 3:**
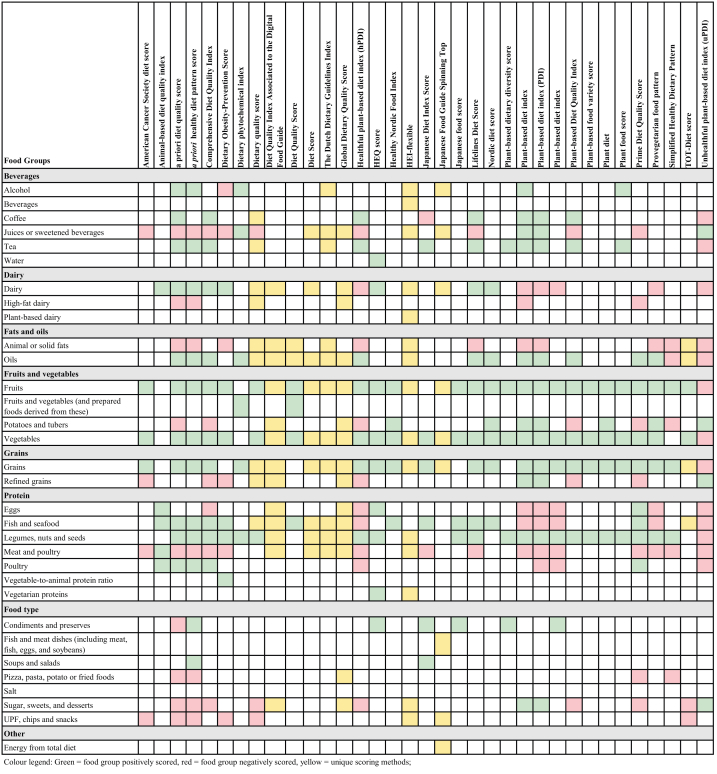
Food groups included in the 35 unique plant-based diet quality indices. Modified from [69].

The number and types of food groups included in the 35 plant-based diet quality indices varied considerably (Table 1). This ranged from 4 food groups in the American Cancer Society diet score [41] and the diet quality score [50], to 33 food groups in the *a priori* diet quality score [43]. The average number of included food groups per index was 12, and most indices had 6 or 7 food groups (*n* = 9) [18,42,45,56,59,60,63,68,69]. Figure 3 outlines the food groups used across the 35 plant-based diet quality indices. The most common overall food groups were vegetables (*n* = 32); fruits (*n* = 32); grains (*n* = 30); and legumes, nuts and seeds (*n* = 27).

Seventeen indices divided food groups into subcategories [[13], [14], [15],[42], [43], [44], [45],^49^,^54^,^55^,^58^,^61^,^65^,^68^]. This included 8 indices that differentiated whether food groups were of plant or animal origin [[13], [14], [15],^42^,^65^], of which 5 further categorized the food groups into healthy and less healthy plant or animal foods [15,42]. For example, the PDI, hPDI, and uPDI by Satija et al. [15] divided 18 food groups into a healthy plant group, a less healthy plant group, and an animal group. In addition to these, 1 index was solely comprised of healthy and unhealthy animal foods [42]. This index was included in the review as it was used in combination with other plant-based diet quality indices, such as the Comprehensive Diet Quality Index [42], to highlight independent associations on the basis of the quality of animal foods.

Calculation methods used to score food group intakes varied significantly across the indices (Table 1). Eighteen indices used [[13], [14], [15],^18^,^42^,[44], [45], [46],^51^,^58^,^61^,^62^,^65^,^67^] population-specific percentile cutoffs (such as tertiles) to calculate the food group scores, with 7 indices using percentile cutoffs that were specific to sex [14,42,46,58,62]. Thirteen (of 35) indices used normative cutoffs independent of population intake [[48], [49], [50],[52], [53], [54], [55], [56],^59^,^60^,^63^,^66^,^69^]. For example, the Dutch Dietary Guidelines Index [53] that is based on epidemiological evidence between foods and diet-related chronic diseases as well as the Dutch Dietary Guidelines, used normative cutoff values for the recommended intake of each food group to calculate the individual food group and total scores. Of the 13 indices that used normative cutoffs, 1 used cutoffs specific to sex, age, and 2 levels of physical activity [59], and 1 used sex-specific cutoffs for just 1 of the food groups [69]. In addition to these, 3 indices used a combination of both normative cutoffs and population-specific percentile cutoffs [18,41,68]. Nine indices used EI adjustments in the calculation of the score [14,42,46,47,49,61,68], for example, the Lifelines Diet Score [61], which calculated intake of the food groups in g/1000 kcal to account for differences in EI between participants, and 23 indices used total EI in their analysis models [[13], [14], [15],^18^,[41], [42], [43], [44], [45], [46],[53], [54], [55],[58], [59], [60],^62^,^65^,^68^]. All indices followed a positive overall scoring system, where the higher the index score the higher the adherence to the dietary pattern. The maximum total score varied from 6 in the Healthy Nordic Food Index [18] to 132 in the *a priori* diet quality score [43].

As outlined in Figure 3, 10 indices comprised of food groups that were all positively scored [18,42,47,56,60,62,63,66,67,68] and 16 indices used a combination of positively and negatively scoring food groups [[13], [14], [15],[41], [42], [43], [44], [45], [46],^55^,^58^,^61^,^65^]. Nine indices scored food groups using unique scoring methods [49,[52], [53], [54],^57^,^59^] such as proportional scoring if exceeding or falling short of the recommended servings [59]. Twenty indices differentiated the scoring between healthy and less healthy plant-based foods on the basis of epidemiological evidence, dietary guidelines, or other criteria [15,44,46,49,54,55,57,59,61], resulting in scoring differences for plant origin food groups (Table 1). For example, in the plant-based diet quality index, the score for the healthy plant food groups increases with higher intake, whereas the score for the unhealthy plant food groups decreases as intake increases [42]. As outlined in Figure 3, of the 35 indices, 20 negatively scored or had cutoff intake limits for meat or poultry [14,15,[41], [42], [43], [44], [45], [46],^49^,[52], [53], [54], [55],^57^,^58^,^61^,^65^]. For example, the PDI, hPDI, and uPDI by Satija et al. [15] all negatively scored meat or poultry, but the Prime Diet Quality Score [55], *a priori* healthy diet pattern score [44], *a priori* diet quality score [43], and the Comprehensive Diet Quality Index score [42] reverse scored meat but positively scored poultry. For dairy foods, 7 indices negatively scored dairy food groups, and 2 other indices differentiated dairy scoring by its fat content, resulting in positively scoring low-fat dairy and reverse scoring high-fat dairy [43,44]. The least represented food groups included water [56] and ultraprocessed foods (UPFs) [46], which were both only included in 1 index each.

### Validation of plant-based diet quality indices

As summarized in Table 2, 30 indices (of 35) used construct validity, criterion validity, and/or reliability in the development of the index. Of the 35 unique indices, 14% (*n* = 5) included index validation as part of their study objectives. Construct validity of the plant-based diet quality indices was assessed for 26 indices [14,15,18, [41], [42], [43],[45], [46], [47], [48],^50^,^52^,^54^,^55^,^57^,^59^,[60], [61], [62],^65^,[67], [68], [69]]. Twenty-five indices reported adequate evidence for construct validity [14,15,18,[41], [42], [43], [44], [45], [46], [47], [48], [49], [50],^52^,^54^,^55^,^57^,[60], [61], [62],^65^,[67], [68], [69]], and the Japanese Food Guide Spinning Top index reported both adequate and inadequate evidence for construct validity [59]. Criterion validity was reported for 5 plant-based diet quality indices [14,15,62], and 4 found adequate evidence for correlations with other validated diet quality indices, including Mediterranean diet scores, DASH, and AHEI scores. Twenty plant-based diet quality indices assessed reliability of the index, with 19 indices reporting adequate reliability [[13], [14], [15],^18^,^42^,^43^,^46^,^48^,^49^,^52^,^55^,^57^,^59^,^61^,^65^,^66^,^67^], and 1 index reporting inadequate reliability [68].

### Modification to plant-based diet quality indices

Of the 106 articles that applied the indices in an independent sample, most index applications were published in 2021 and 2022 (Figure 2). The most commonly used plant-based diet quality indices were the hPDI (*n* = 62), PDI (*n* = 49), and uPDI (*n* = 49) [15], and the provegetarian food pattern (*n* = 18) [14] (Supplemental Table 2). Approximately 53% (*n* = 114) of all application of the indices were modified versions of the unique plant-based diet quality indices. As shown in Supplemental Table 2, most articles adapted the indices because of limitations in the dietary intake data available (19%, *n* = 41) or to make the index more culturally applicable to the study population (15%, *n* = 33).

## Discussion

This scoping review identified a total of 35 unique plant-based diet quality indices, over half of which were published in the last 5 years. Although the development, methodology, and validation of these indices varied considerably, some common features were apparent. Sixteen plant-based diet quality indices were developed to reflect well-established epidemiological associations between plant and animal foods and health outcomes, and 20 indices differentially scored healthy and less healthy plant-based foods. Thirty indices assessed either construct validity, criterion validity, or the reliability of the index. When examining the 106 articles that used these indices and cultural and methodological modifications to the food groups and calculation methods were widespread. Based on these findings, researchers should consider 3 key criteria when selecting a plant-based diet quality index: *1*) the research question aligns with a basis for development of either epidemiological evidence, dietary guidelines, or a traditional dietary pattern; *2*) the index methodology and researcher’s dietary assessment tool for constructing food groups allows differentiation of healthy and unhealthy plant foods; and *3*) the index has been validated for use in a comparable population.

The majority of the plant-based diet quality indices identified in this review were developed to reflect either epidemiological evidence for associations between plant and animal foods and health outcomes or plant-based dietary guidelines. A recent review of the construction of *a priori* diet quality indices found that indices based on epidemiological research were more likely to provide detailed food group scoring methodologies compared with indices developed based on dietary guidelines [37]. This was attributed to a difference in the purpose of the indices, where those that aimed to assess dietary intake against dietary guidelines to guide public health promotion usually included a broader range of food groups as a result. The present review supports these findings, where plant-based diet quality indices based on epidemiological research, such as the *a priori* healthy diet pattern score [44], included specific food groups such cruciferous vegetables, whereas indices based on dietary guidelines, such as the dietary quality score [48], included broader food groups, such as fruits and vegetables. Thus, the basis for development for plant-based diet quality indices is likely to directly impact on the design and applicability of the index for use in future research and should be a key consideration for researchers.

Almost half of the plant-based diet quality indices differentiated the scoring between healthy and less healthy plant-based foods. This is consistent with recommendations for the development of diet quality indices in general, where food groups should be separately scored on the basis of whether they are encouraged or discouraged to optimize health and reduce risk of chronic disease [28]. Categorizing plant-based foods into healthy and less healthy groups offers specific advantages, including the estimation of separate indices, as shown by the PDI, hPDI, and uPDI [15], and the identification of specific plant foods that researchers and policy makers should target to improve the diet quality and possibly the food systems in their population [28]. Although it was common among the indices in this review to differentiate the healthiness of foods such as grains and meat, only 1 index scored intake of UPFs [46]. Considering the growing evidence of the negative health impacts from overconsumption of UPFs [70] and the increasing availability of plant-based meat alternatives classified as UPFs [71], this is an important emerging area for research and an opportunity for plant-based diet quality indices to better account for these both healthy and less healthy plant-based foods in their scoring methodologies.

The scoring methods used to derive the plant-based diet quality indices varied considerably across studies. Eighteen indices, such as the Nordic diet score [62] and plant-based diet quality index [42], used percentile cutoffs based on population-specific intakes, such as tertiles or quintiles. Although this is not uncommon for diet quality methodologies [72], this approach has several limitations. Most notably, cutoffs developed within particular populations are specific to those populations, and therefore, their applicability to other populations is limited. This reduces our ability to make comparisons on the consumption of plant-based foods between countries, which is important for consistent use and interpretation of indices [73,74]. To overcome this, researchers should report the absolute food group intakes overall and by any divisions or subgroups used, such as quintiles, so that even if the results are population-specific, the intakes can still be compared. Indices such as the Japanese Food Guide Spinning Top [59] and the Dutch Dietary Guidelines Index [53] scored food groups on the basis of targets for recommended number of servings, or normative cutoffs based on levels of intake considered to be healthy. Although the scoring of food groups on the basis of normative cutoffs has the advantage of providing insights into beneficial or harmful effects on the body [28], low intakes of food groups in some populations may limit the index from discriminating well between food components and the total score. This is particularly apparent for foods such as legumes, which are not widely consumed in Western countries [[75], [76], [77]] such as in Australia where the per capita yearly intake of 2.9 kg is much lower than the worldwide average of 5 kg [78]. Moreover, a previous review of *a priori* diet quality indices highlights that cutoff values should be specific to age, sex, weight, and PALs owing to differences for total nutrient requirements [37]. However, in this review, the majority of indices only used sex-specific cutoffs. Although this would still result in more specific scoring than no subgroup scoring at all, the use of further specific cutoff values would increase the precision of the index scoring, which would have implications for the final outcomes in these articles. Additionally, most indices weighted each food group equally in the final score, which may not adequately capture whether food groups differentially impact on health [74]. Although there may not be sufficient evidence to weight all food groups differently in the construction of an index, some indices did consider this. For example, the American Cancer Society diet score [41] weighted the scoring for fruit and vegetables differently on the basis of national recommendations. Half of the score was based on the intake of fruits or vegetables, and the other half was based on the variety of fruits or vegetables being consumed. Additionally, the role and use of total EI also varied across the indices, with roughly a quarter of the indices using EI adjustments to calculate the score and two-thirds adjusting for EI in subsequent analytical models examining the index and the specific outcomes. For example, indices such as Diet Quality Index Associated to the Digital Food Guide [49] and the plant food score [68] calculate a score on the basis of food intake per 1000 kcal and hence assess the proportion of that food in the whole diet [17]. This is in comparison to indices such as Simplified Healthy Dietary Pattern [45] and the Nordic diet score [62], which do not use EI in the score calculation and rather assess the quantity of the different foods in the diet. Thus, researchers should not only consider the food groups included in the index but also the calculation method and the use of EI when applying an index in their specific study context.

This review demonstrated that about half of the plant-based diet quality indices had been modified when they were applied in subsequent studies. These studies aimed to apply the plant-based diet quality index but often did not always have the most appropriate dietary intake data and therefore had to adjust the calculation of the established index. The majority of modifications were based on either cultural adaptation of the study population or because of limitations in the available dietary data. This was often because of the dietary assessment method not collecting a specific food group, because of differences in food culture [79], or the dietary intake data may not have been specific enough, such as the kind of fat the margarine was made from [80]. For example, Kawasaki et al. [81] modified the hPDI and uPDI by including foods such as seaweeds and soy products to better reflect the Japanese diet of the study population, whereas Kim et al. [82] modified the provegetarian food pattern by excluding vegetable oil as the dietary intake data only assessed margarine consumption. This is consistent with a recent review of diet quality indices, where approximately one-third of all indices had been modified, mostly because of limitations in the dietary intake data or so that the index was more suitable for the population being studied [83]. Although we identified that some modifications to the original index were minor, some studies adapted all the food groups in the index, such as in Noruzi et al. [84]. In instances such as this, the index is likely to be very different to the original publication, and therefore, validation of the modified version of the index is most likely required. Additionally, as previous reviews have also identified [21], indices that have been tested in specific populations, such as the plant-based food variety score [66], which only included women in the study population [54]; hence, the analysis has limited generalizability to other populations. Thus, given the variability in the number of indices that have been validated and the methods used for validation, researchers should ensure that the index has been validated in a way that is appropriate for their application. Although there is no gold standard for validating an *a priori* diet quality index, researchers should consider how the index validation methods are relevant for their use and study context and acknowledge any limitations. Furthermore, because some indices in this review did not report a reason for modifying the index, more consistent reporting of adaptations are needed so that the evidence for plant-based diet quality indices can be more accurately synthesized and used for guideline development.

A strength of this scoping review was the systematic search methodology undertaken and the use of the PRISMA-ScR reporting guidelines. A further strength to this review was the examination of the methods used by researchers to develop and validate their index, which is an important consideration for others when assessing the suitability of an index for their study. As plant-based diet quality research is an emerging field, another strength was updating the review that found 54 additional articles that had been published and met the inclusion criteria in the time between searches. However, there is also the possibility that some indices have been missed because of the extensive body of literature and frequency with which new indices are being published. Nonetheless, the potential for this was minimized by our use of broad search terms, piloting of the search terms and search results, and reference list searching. Furthermore, the search terms were developed on the basis of known indices in the literature, previous reviews of diet quality indices, and general terms used about plant-based diets in research. Another limitation of this scoping review is defining what constitutes a new index, rather than a modification of an already published index. For consistency, plant-based diet quality indices were defined as a new index if they did not reference another plant-based diet quality index or the article was published as an index development article. Additionally, although this review focused on food-based indices, it is important to recognize there are a several indices which would be considered plant-based in that they assess a plant-based dietary pattern but do so using nutrients or lifestyle factors as part of the score, which was out of scope for this review. For example, the Paleolithic Diet Score, and the Dutch Healthy Diet index could both be considered plant-based diet quality indices but were excluded as they contain calcium and sodium components as part of the index [85,86]. Additionally, the Vegetarian Lifestyle Index by Le at al. [87] contained food group components as well as components for daily exercise and sunlight exposure, so it was excluded. Although indices reflecting the Mediterranean diet were excluded because of feasibility reasons, this was considered an acceptable limitation because of the extensive literature reviewing these indices [21,22,30]. Moreover, this exclusion did not prohibit us from addressing the aims of a scoping review, which is to determine the scope of the literature and provide detail on volume of publications and focus of the topic [19]. Lastly, detailed strengths and limitations of each index was not possible within the context of this scoping review because of the large number of indices identified. Nonetheless, some consideration of strengths and limitations of the index are captured by our critical review of the indices basis for development, methodology, and validation.

The results of this research have implications for future plant-based diet research. This review provides a summary of published plant-based diet quality indices and a comparison of their development, methodology, and validity. These 3 criteria are important for researchers to consider when choosing the most suitable index according to their study aims. For example, for researchers interested in using an index that differentially scores healthy and nonhealthy plant foods on the basis of epidemiological evidence, we have identified 13 plant-based diet quality indices that could be used (Table 3) [15,42,44,45,46,[52], [53], [54], [55],^61^]. In contrast, if a researcher intends to use a plant-based index that can be constructed from a short FFQ (≤12 items) and is developed on the basis of dietary guidelines then only 5 indices meet these criteria [50,52,59,61,69]. Future research should consider the role of processing in the development of new plant-based diet quality indices, to better reflect the emerging literature on the association between degree of processing, healthiness of diets and health outcomes [[88], [89], [90]]. In addition, more consistent and detailed reporting and validation of these indices is also recommended to aid with evidence synthesis. Further research on the balance of plant and animal foods is important for the development of food-based dietary guidelines, and plant-based diet quality indices play a key role in developing this knowledge base.

**TABLE 3:** Overview of the basis for development, methodology, and validity of the 35 plant-based diet quality indices

Index name	Basis for development				Methodology			Validity
	Other diet quality indices	Epidemiological evidence	Dietary guidelines	Traditional diet	Score differentiated for healthy vs. less healthy plant-based foods	Requires data on ≤12 food groups	Percentile cutoffs	Construct, criteria, or reliability
American Cancer Society diet score [40]					x	x		x
Animal-based diet quality index (aDQI) [41]	x	x				x	x	x
*apriori* diet quality score [42]	x				x		x	x
*apriori* healthy diet pattern score [43]		x			x		x	
Comprehensive Diet Quality Index (cDQI) [41]	x	x			x		x	x
The Dietary Obesity-Prevention Score (DOS) [45]		x			x		x	x
Dietary phytochemical index [46]						x		x
Dietary quality score [47]			x		x			x
Diet Quality Index Associated to the Digital Food Guide (DQI-DFG) [48]				x	x	x		x
Diet Quality Score [49]	x		x			x		x
Diet Score [51]		x	x		x	x		x
The Dutch Dietary Guidelines (DDG) Index [52]		x	x		x			
Global Diet Quality Score (GDQS) [53]	x	x			x			x
Healthful plant-based diet index (hPDI) [15]	x	x			x		x	x
Healthy Eating Quiz (HEQ) score [55]	x					x		
Healthy Nordic Food Index [18]		x		x		x	x	x
HEI-flexible (HEI-flex) [56]	x		x		x			x
Japanese Diet Index Score [57]	x			x		x	x	
Japanese Food Guide Spinning Top [58]			x		x	x		x
Japanese food score [59]	x			x		x		x
Lifelines Diet Score (LLDS) [60]		x	x		x	x	x	x
Nordic diet score [61]				x		x	x	x
Plant-based dietary diversity score [62]	x					x		
Plant-based diet index (PDI) [13]	x		x	x			x	x
PDI [15]	x	x			x		x	x
PDI [64]						x	x	x
Plant-based Diet Quality Index (pDQI) [41]	x	x			x	x	x	x
Plant-based food variety score (PFVS) [65]	x					x		x
Plant diet [66]						x	x	x
Plant food score (PFS) [67]						x		x
Prime Diet Quality Score [PDQS] (54)		x			x			x
Provegetarian food pattern [14]		x				x	x	x
Simplified Healthy Dietary Pattern [44]		x			x	x	x	x
TOT-Diet score [68]			x		x	x		x
Unhealthful plant-based diet index (uPDI) [15]	x	x			x		x	x
Total	*n* = 16 46%	*n* = 16 46%	*n* = 9 26%	*n* = 6 17%	*n* = 20 57%	*n* = 22 63%	*n* = 18 51%	*n* = 30 86%

This review highlights the diversity of approaches used in the development, scoring and validation of plant-based diet quality indices in recent decades. Although the indices varied substantially, common features such as the use of epidemiological evidence for associations between diet and health outcomes for the basis of development, the use of normative or percentile cutoffs, and the application of construct validity were all commonly described in the publications. For future use of plant-based diet quality indices, researchers should not only pay attention to the food group components in the index but also to the basis for their development, the methods used to calculate an index score and how and in what population the index was validated to determine which index is most appropriate for use in their study. By providing a summary of these development approaches, this review can be used to inform future development, refinement, and reporting of plant-based diet quality indices.

## Acknowledgments

We acknowledge the liaison librarians from Deakin University for their support of this research. The authors’ responsibilities were as follows—all authors: developed the research plan and contributed to the screening of articles; LEM: developed the search strategy; LEM: extracted the data; KMD: verified the extraction; LEM: wrote the first draft of the manuscript; and all authors: developed the research plan, contributed to the screening of articles and a critical review of the manuscript, and read and approved the final manuscript.

## Funding

LEM is supported by a Deakin University Postgraduate Research Scholarship and a CSIRO R+ top-up scholarship. KMD receives support from the Foundation for High Blood Pressure Research. KML is supported by a National Health and Medical Research Council Emerging Leadership Fellowship (APP1173803).

## Author disclosures

The authors report no conflicts of interest. SAM is an Editor of *Current Developments in Nutrition* and played no role in the Journal’s evaluation of the manuscript.

## Data Availability

Data described in the manuscript, code book, and analytic code will be made available upon request pending application and approval.

## Appendix A Supplementary data

Multimedia component1 Multimedia component1
