# Supplementary material for: A scoping review of approaches used to develop plant-based diet quality indices
Source: Curr Dev Nutr. 2023 Feb 28;7(4):100061. doi: 10.1016/j.cdnut.2023.100061 (PMC10257227; doi:10.1016/j.cdnut.2023.100061)
Supplement: Multimedia component1 [file mmc1.docx]

**Supplementary Material**

**Title:**

A scoping review of approaches used to develop plant-based diet quality indices

**First Author:**

Laura E Marchese

**Supplementary table 1:** Preferred Reporting Items for Systematic reviews and Meta-Analyses extension for Scoping Reviews (PRISMA-ScR) Checklist **(1)**

| **SECTION** | **ITEM** | **PRISMA-ScR CHECKLIST ITEM** | **REPORTED ON PAGE #** |
| --- | --- | --- | --- |
| **TITLE** | | | |
| Title | 1 | Identify the report as a scoping review. | 1 |
| **ABSTRACT** | | | |
| Structured summary | 2 | Provide a structured summary that includes (as applicable): background, objectives, eligibility criteria, sources of evidence, charting methods, results, and conclusions that relate to the review questions and objectives. | 3-4 |
| **INTRODUCTION** | | | |
| Rationale | 3 | Describe the rationale for the review in the context of what is already known. Explain why the review questions/objectives lend themselves to a scoping review approach. | 4-6 |
| Objectives | 4 | Provide an explicit statement of the questions and objectives being addressed with reference to their key elements (e.g., population or participants, concepts, and context) or other relevant key elements used to conceptualize the review questions and/or objectives. | 6 |
| **METHODS** | | | |
| Protocol and registration | 5 | Indicate whether a review protocol exists; state if and where it can be accessed (e.g., a Web address); and if available, provide registration information, including the registration number. | Available upon request |
| Eligibility criteria | 6 | Specify characteristics of the sources of evidence used as eligibility criteria (e.g., years considered, language, and publication status), and provide a rationale. | 6-9 |
| Information sources* | 7 | Describe all information sources in the search (e.g., databases with dates of coverage and contact with authors to identify additional sources), as well as the date the most recent search was executed. | 8-9 |
| Search | 8 | Present the full electronic search strategy for at least 1 database, including any limits used, such that it could be repeated. | 8 |
| Selection of sources of evidence† | 9 | State the process for selecting sources of evidence (i.e., screening and eligibility) included in the scoping review. | 8-9 |
| Data charting process‡ | 10 | Describe the methods of charting data from the included sources of evidence (e.g., calibrated forms or forms that have been tested by the team before their use, and whether data charting was done independently or in duplicate) and any processes for obtaining and confirming data from investigators. | 9-10 |
| Data items | 11 | List and define all variables for which data were sought and any assumptions and simplifications made. | 9-10 |
| Critical appraisal of individual sources of evidence§ | 12 | If done, provide a rationale for conducting a critical appraisal of included sources of evidence; describe the methods used and how this information was used in any data synthesis (if appropriate). | N/A |
| Synthesis of results | 13 | Describe the methods of handling and summarizing the data that were charted. | 9-10 |
| **RESULTS** | | | |
| Selection of sources of evidence | 14 | Give numbers of sources of evidence screened, assessed for eligibility, and included in the review, with reasons for exclusions at each stage, ideally using a flow diagram. | 10-11 and Fig 1 |
| Characteristics of sources of evidence | 15 | For each source of evidence, present characteristics for which data were charted and provide the citations. | 10-15 |
| Critical appraisal within sources of evidence | 16 | If done, present data on critical appraisal of included sources of evidence (see item 12). | N/A |
| Results of individual sources of evidence | 17 | For each included source of evidence, present the relevant data that were charted that relate to the review questions and objectives. | Table 1 and 2 |
| Synthesis of results | 18 | Summarize and/or present the charting results as they relate to the review questions and objectives. | 10-15 |
| **DISCUSSION** | | | |
| Summary of evidence | 19 | Summarize the main results (including an overview of concepts, themes, and types of evidence available), link to the review questions and objectives, and consider the relevance to key groups. | 15-20 |
| Limitations | 20 | Discuss the limitations of the scoping review process. | 19-20 |
| Conclusions | 21 | Provide a general interpretation of the results with respect to the review questions and objectives, as well as potential implications and/or next steps. | 20-21 |
| **FUNDING** | | | |
| Funding | 22 | Describe sources of funding for the included sources of evidence, as well as sources of funding for the scoping review. Describe the role of the funders of the scoping review. | 1 |

JBI = Joanna Briggs Institute; PRISMA-ScR = Preferred Reporting Items for Systematic reviews and Meta-Analyses extension for Scoping Reviews.

* Where *sources of evidence* (see second footnote) are compiled from, such as bibliographic databases, social media platforms, and Web sites.

† A more inclusive/heterogeneous term used to account for the different types of evidence or data sources (e.g., quantitative and/or qualitative research, expert opinion, and policy documents) that may be eligible in a scoping review as opposed to only studies. This is not to be confused with *information sources* (see first footnote).

‡ The frameworks by Arksey and O’Malley (6) and Levac and colleagues (7) and the JBI guidance (4, 5) refer to the process of data extraction in a scoping review as data charting*.*

§ The process of systematically examining research evidence to assess its validity, results, and relevance before using it to inform a decision. This term is used for items 12 and 19 instead of "risk of bias" (which is more applicable to systematic reviews of interventions) to include and acknowledge the various sources of evidence that may be used in a scoping review (e.g., quantitative and/or qualitative research, expert opinion, and policy document).

**Supplementary table 2:** Overview of plant-based diet quality indices and how they have been modified for use in different populations

| **Reference and country** | **Index modification** | | | | | | | **No index modification** | **Outcome(s) assessed** |
| --- | --- | --- | --- | --- | --- | --- | --- | --- | --- |
|  | **Food groups** | | | | **Calculation** | | |  |  |
|  | **Country specific food groups** | **Foods groups modified - not explained why** | **Foods groups modified due to dietary assessment tool data** | **Food groups modified - as per previous studies** | **Scoring modified due to cohort consumption** | **Scoring of food groups** | **Total score range** |  |  |
| **Animal-based diet quality index (aDQI) (n=1)** (2) | | | | | | | | | |
| Brunin et al. France 2022 (3) |  |  |  |  |  |  |  | x | Dietary patterns, organic food consumption and socio-demographics |
| ***a priori* diet quality score (n=1)** (4) | | | | | | | | | |
| Gray et al. 2022 US (5) |  |  |  |  |  |  |  | x | Nonalcoholic fatty liver disease |
| **Comprehensive Diet Quality Index (n=1)** (2) | | | | | | | | | |
| Brunin et al. France 2022 (3) |  |  |  |  |  |  |  | x | Dietary patterns, organic food consumption and socio-demographics |
| **Dietary phytochemical index (n=7)** (6) | | | | | | | | | |
| Amirkhizi et al. Iran 2022 (7) |  | x |  |  |  |  |  |  | Knee osteoarthritis |
| Asgari et al. Iran 2021 (8) |  | x |  |  |  |  |  |  | General and central obesity |
| Bahadoran et al. Iran 2013 (9) |  | x |  |  |  |  |  |  | Cardiometabolic risk factors |
| Esmaeily et al. Iran 2022 (10) |  |  |  |  |  |  |  | x | ApoA2–256T > C, ghrelin and leptin hormones, and biochemical markers |
| Ghoreishy et al. Iran 2021 (11) | x |  |  |  |  |  |  |  | Breast cancer |
| Mirzababaei et al. Iran 2022 (12) |  | x |  |  |  |  |  |  | Resting metabolic rate |
| Noruzi et al. Iran 2022 (13) |  | x |  |  |  |  |  |  | Circadian rhythm and mental health disorders |
| **Diet Quality Index Associated to the Digital Food Guide (n=1)** (14) | | | | | | | | | |
| Assumpção et al. Brazil 2022 (15) |  |  |  |  |  |  |  | x | Brazilian Healthy Eating Index-Revised |
| **Diet Quality Score (n=1)** (16) | | | | | | | | | |
| Bhattacharya et al. UK 2021 (17) |  | x |  |  |  | x | x |  | Prevalence of clonal hematopoiesis and adverse cardiovascular events |
| **Global Dietary Quality Score (n=4)** (18) | | | | | | | | | |
| Angulo et al. Mexico 2021 (19) |  |  | x |  |  |  | x |  | Concurrent weight and waist circumference change |
| Birk et al. India 2021 (20) |  |  | x |  |  |  | x |  | Prediabetes |
| Fung et al. US 2021 (21) |  |  |  |  |  | x |  |  | Risk of type 2 diabetes |
| He et al. China 2021(22) |  |  |  |  |  |  |  | x | Nutrient adequacy and metabolic syndrome |
| **Healthful plant-based diet index (hPDI) (n=62)** (23) | | | | | | | | | |
| Abaj et al. Iran 2022 (24) |  |  |  |  |  |  |  | x | Caveolin‐1, metabolic factors, serum inflammatory markers and anthropometric measures |
| Aljuraiban et al. Saudi Arabia 2022 (25) |  |  |  |  |  | x | x |  | High-sensitivity C-reactive protein level |
| Aljuraiban Saudi Arabia 2022 (26) |  |  |  |  |  | x | x |  | Psychological stress |
| Amini et al. Iran 2021 (27) |  |  |  |  |  | x | x |  | Metabolic syndrome |
| Anyene et al. US 2021 (28) |  |  |  |  |  |  |  | x | Breast cancer recurrence and survival |
| Baden et al. US 2019 (29) |  |  |  |  |  |  |  | x | Total and cause-specific mortality |
| Baden et al. US 2019 (30) |  |  |  |  |  |  |  | x | Plasma adiposity-associated biomarker concentrations |
| Baden et al. US 2020 (31) |  |  |  |  |  |  |  | x | Health-related quality of life |
| Bolori et al. Iran 2019 (32) |  |  |  |  |  |  |  | x | Levels of liver enzymes, inflammatory factors and adipocytes profile |
| Brunin et al. France 2022 (3) |  |  |  |  |  |  |  | x | Dietary patterns, organic food consumption and socio-demographics |
| Carto et al. US 2022 (33) |  |  |  | x |  |  | x |  | Erectile dysfunction |
| Chen et al., Singapore 2018 (34) |  |  | x |  | x |  |  |  | Type 2 diabetes |
| Chen et al., US 2021 (35) |  |  |  |  |  |  |  | x | Risk of type 2 diabetes |
| Chen et al. US 2022 (36) |  | x |  |  |  |  | x |  | Serum metabolite profile and incident dieabetes |
| Daneshzad et al. Iran 2020 (37) |  |  |  |  |  | x | x |  | Sleep status and mental health |
| Flores et al. US 2021 (38) |  |  |  |  |  |  |  | x | Risk of developing diabetes |
| Gehring et al. France 2020 (39) |  |  |  |  |  |  |  | x | Consumption of UPFs |
| Gómez-Donozo et al. Spain 2019 (40) |  |  |  |  |  |  |  | x | Overweight/obesity incidence |
| González-Ortiz et al. Mexico 2021 (41) | x |  |  |  |  |  | **x** |  | Nutritional status, hyperkalaemia, energy/protein intake, serum potassium levels, or MIS |
| Grisotto et al. US 2022 (42) |  |  |  |  |  |  |  | **x** | Early onset of natural menopause |
| Heianza et al. UK 2020 (43) |  |  | x |  |  |  |  |  | Risk of CVD |
| Heianza et al. UK 2021 (44) |  |  | x |  |  |  |  |  | Risks of obesity and related cardiovascular abnormalities |
| Kawasaki et al. Japan 2021 (45) | x |  |  |  |  | x | x |  | Chronotype |
| Kim et al. South Korea 2020 (46) | x |  | x |  |  |  | x |  | Incident metabolic syndrome |
| Kim et al. South Korea 2021 (47) | x |  | x |  |  |  | x |  | Risk of hypertension |
| Kim et al. US 2019 (48) |  |  | x |  |  |  | x |  | Incident Cardiovascular Disease, Cardiovascular Disease Mortality, and All-Cause Mortality |
| Kim et al. US 2020 (49) |  |  | x |  |  |  | x |  | Risk of incident hypertension |
| Kim et al. South Korea 2021 (50) | x |  | x | x |  |  | x |  | Risk of total and disease-specific mortality |
| Kim et al. South Korea 2022 (51) | x |  | x | x |  |  | x |  | Risk of type 2 diabetes |
| Kouvari et al. Greece 2022 (52) | x |  |  |  |  |  | x |  | Fatal/non-fatal cardiovascular disease incidence |
| Laouali et al. France 2021 (53) |  |  |  |  |  |  |  | x | Type 2 diabetes and hypertension risks |
| Lee et al. Korea 2021 (54) | x |  | x |  |  |  | x |  | Risk of dyslipidemia, and individual lipid disorders |
| Li et al. US 2021(55) |  |  |  |  |  |  |  | x | Gut microbiome, and cardiometabolic risk markers. |
| Li et al. US 2022 (56) |  |  |  |  |  |  |  | x | Mortality risk |
| Liang et al. China 2022 (57) | x |  |  |  |  |  | x |  | Cognitive impairment |
| Lim et al. Singapore 2022 (58) |  |  | x |  |  | x |  |  | Fecundability |
| Loeb et al. US 2022 (59) |  |  |  |  |  |  |  | x | Prostate cancer risk |
| Merino et al. UK, US 2021 (60) |  |  | x |  |  |  | x |  | Risk and severity of COVID-19 |
| Mohamadi et al. Iran 2022 (61) |  |  |  |  |  | x | x |  | Metabolically healthy obesity and metabolically unhealthy obesity phenotypes |
| Mousavi et al. Iran 2021 (62) |  |  |  |  |  |  |  | x | Risk of glioma |
| Mousavi et al. Iran 2022 (63) |  |  |  |  |  |  |  | x | Psychological disorders |
| Mouzannar et al. US 2021 (64) |  |  |  | x |  |  | x |  | Prostate specific antigen levels |
| Oncina‐Cánovas et al. Spain 2022 (65) |  | x |  |  |  |  | x |  | Cardiometabolic risk |
| Pourreza et al. Iran 2021 (66) |  |  |  |  |  |  | x |  | Sleep quality and inflammatory markers |
| Ratjen et al. Germany 2020 (67) |  |  |  |  |  |  |  | x | Measures of abdominal fat distribution and liver fat content. |
| Ratjen et al. Germany 2021 (68) |  |  |  |  |  |  |  | x | All-cause mortality |
| Romanos-Nanclares et al. Spain 2020 (69) | x |  |  |  |  |  | x |  | Breast Cancer incidence |
| Salomé et al. France 2021 (70) |  |  |  |  |  |  |  | x | Food processing, patterns of protein intake, and cardiometabolic risk. |
| Satija et al. US 2019 (71) |  |  |  |  |  |  |  | x | Weight change |
| Shahavandi et al. Iran 2020 (72) |  |  |  |  |  |  |  | x | Visceral adiposity, lipid accumulation product, and triglyceride-glucose index |
| Shan et al. US 2020 (73) |  |  |  |  |  |  |  | x | Risk of incident CVD |
| Shirzadi et al. Iran 2022 (74) |  | x |  |  |  | x | x |  | CVD risk factors |
| Stanford et al. Australia 2021 (75) |  | x |  |  |  |  | x |  | Uremic Toxins, and Gut Microbiota |
| Wang et al. Australia 2021 (76) |  |  |  |  |  | x | x |  | Risk of obesity |
| Waterplas et al. Belgium 2020 (77) |  |  |  |  |  |  |  | x | Anthropometric parameters and blood lipids |
| Weber et al. Germany 2022 (78) |  |  |  |  |  |  |  | x | Plasma boron concentrations |
| Weston et al. US 2022 (79) | x |  |  | x |  |  |  |  | Incident cardiovascular disease and all-cause mortality |
| Wu et al. Singapore 2019 (80) |  |  | x |  |  |  | x |  | Cognitive impairment in later life |
| Yue et al. US 2021 (81) |  |  |  |  |  |  |  | x | Risk of colorectal cancer |
| Zagarins et al. US 2021 (82) |  |  |  |  |  |  |  | x | Weight- related outcomes |
| Zamani et al. Iran 2020 (83) |  |  |  |  |  | x | x |  | Psychological profile and obesity |
| Zhou et al. Singapore 2021 (84) |  |  | x |  |  |  | x |  | Healthy ageing |
| **Healthy Nordic Food Index (n=6)** (85) | | | | | | | | | |
| Gunge et al. Denmark 2017 (86) |  |  |  |  |  |  |  | x | Risk of myocardial infarction |
| Jensen et al. Norway 2018 (87) | x |  | x |  |  |  |  |  | Energy-adjusted dietary factors and lifestyle factors |
| Li et al. Sweden 2015 (88) |  |  |  |  |  |  |  | x | Risk of breast cancer |
| Noruzi et al. Iran 2021 (89) | x |  | x |  |  |  |  |  | Metabolic Syndrome and Obesity |
| Roswall et al. Denmark, Germany, Italy, Netherlands, United Kingdom 2014 (90) |  |  | x |  |  |  | x |  | Changes in weight and waist circumference |
| Roswall et al. Sweden 2015 (91) |  |  |  |  |  |  |  | x | Dietary composition, micronutrient density, lifestyle, socioeconomic factors |
| **Japanese Diet Index Score (n=1)** (92) | | | | | | | | | |
| Lu et al. Japan 2021 (93) |  |  |  | x |  |  | x |  | Incident dementia |
| **Japanese Food Guide Spinning Top (n=2)** (94) | | | | | | | | | |
| Kurotani et al. Japan 2020 (95) |  |  |  |  |  | x |  |  | Association with age, sex, household income, and Japanese dietary guidelines |
| Watanabe et al. Japan 2022 (96) |  | x |  |  |  |  | x |  | Prevalence of physical and comprehensive frailty |
| **Lifelines Diet Score (LLDS) (n=3)** (97) | | | | | | | | | |
| Cai et al. Netherlands 2021 (98) |  |  |  |  |  |  |  | x | Incident chronic kidney disease or eGFR decline |
| Moazzen et al. Netherlands 2022 (99) |  |  |  |  |  |  | x |  | Risk of gastrointestinal cancer |
| Sohouli et al. Iran 2022 (100) |  |  |  |  |  | x | x |  | Risk of breast cancer |
| **Nordic diet score (n=3)** (101) | | | | | | | | | |
| Bonaccio et al. Italy 2021 (102) |  |  |  |  | **x** |  | **x** |  | All-casue and cause-specific mortality |
| Esposito et al. Italy 2022 (103) |  |  |  |  |  |  | **x** |  | Biological ageing |
| Grosso et al. Italy 2020 (104) |  |  |  |  |  |  |  | **x** | Impact on resources use and greenhouse gas emissions |
| **Plant-based dietary diversity score (n=1)** (105) | | | | | | | | | |
| Liu et al. China 2022 (106) |  |  |  |  |  |  |  | x | Cognitive impairment |
| **Plant-based diet index (PDI) (n=49)** (23) | | | | | | | | | |
| Abaj et al. Iran 2022 (24) |  |  |  |  |  |  |  | x | Caveolin‐1, metabolic factors, serum inflammatory markers and anthropometric measures |
| Aljuraiban et al. Saudi Arabia 2022 (25) |  |  |  |  |  | x | x |  | High-sensitivity C-reactive protein level |
| Aljuraiban Saudi Arabia 2022 (26) |  |  |  |  |  | x | x |  | Psychological stress |
| Amini et al. Iran 2021 (27) |  |  |  |  |  | x | x |  | Metabolic syndrome |
| Anyene et al. US 2021 (28) |  |  |  |  |  |  |  | x | Breast cancer recurrence and survival |
| Baden et al. US 2019 (29) |  |  |  |  |  |  |  | x | Total and cause-specific mortality |
| Baden et al. US 2019 (30) |  |  |  |  |  |  |  | x | Plasma Adiposity-Associated Biomarker Concentrations |
| Baden et al. US 2020 (31) |  |  |  |  |  |  |  | x | Health-related quality of life |
| Bolori et al. Iran 2019 (32) |  |  |  |  |  |  |  | x | Levels of liver enzymes, inflammatory factors and adipocytes profile |
| Brunin et al. France 2022 (3) |  |  |  |  |  |  |  | x | Dietary patterns, organic food consumption and socio-demographics |
| Carto et al. US 2022 (33) |  |  |  | x |  |  | x |  | Erectile dysfunction |
| Chen et al., Singapore 2018 (34) |  |  | x |  | x |  |  |  | Type 2 diabetes |
| Chen et al., US 2021 (35) |  |  |  |  |  |  |  | x | Risk of type 2 diabetes |
| Daneshzad et al. Iran 2020 (37) |  |  |  |  |  | x | x |  | Sleep status and mental health |
| Flores et al. US 2021 (38) |  |  |  |  |  |  |  | x | Risk of developing diabetes |
| González-Ortiz et al. Sweden 2020 (107) |  | x |  | x |  |  | x |  | Measures of insulin sensitivity and inflammation |
| Grisotto et al. US 2022 (42) |  |  |  |  |  |  |  | **x** | Early onset of natural menopause |
| Kim et al. South Korea 2020 (46) | x |  | x |  |  |  | x |  | Incident metabolic syndrome |
| Kim et al. South Korea 2021 (47) | x |  | x |  |  |  | x |  | Risk of hypertension |
| Kim et al. US 2019 (48) |  |  | x |  |  |  | x |  | Incident cardiovascular disease, cardiovascular disease mortality, and all-cause mortality |
| Kim et al. US 2020 (49) |  |  | x |  |  |  | x |  | Risk of incident hypertension |
| Kim et al. South Korea 2021 (50) | x |  | x | x |  |  | x |  | Risk of total and disease-specific mortality |
| Kim et al. South Korea 2022 (51) | x |  | x | x |  |  | x |  | Risk of type 2 diabetes |
| Kouvari et al. Greece 2022 (52) | x |  |  |  |  |  | x |  | Fatal/non-fatal cardiovascular disease incidence |
| Laouali et al. France 2021 (53) |  |  |  |  |  |  |  | x | Type 2 diabetes and hypertension risks |
| Lazarova et al. Canada 2022 (108) |  |  |  |  |  | x |  |  | Cardiovascular disease risk |
| Lee et al. Korea 2021 (54) | x |  | x |  |  |  | x |  | Risk of dyslipidemia, and individual lipid disorders |
| Li et al. US 2022 (56) |  |  |  |  |  |  |  | **x** | Mortality risk |
| Liang et al. China 2022 (57) | x |  |  |  |  |  | x |  | Cognitive impairment |
| Lim et al. Singapore 2022 (58) |  |  | x |  |  | x |  |  | Fecundability |
| Loeb et al. US 2022 (59) |  |  |  |  |  |  |  | x | Prostate cancer risk |
| Mousavi et al. Iran 2021 (62) |  |  |  |  |  |  |  | x | Risk of glioma |
| Mousavi et al. Iran 2022 (63) |  |  |  |  |  |  |  | x | Psychological disorders |
| Mouzannar et al. US 2021 (64) |  |  |  | x |  |  | x |  | Prostate specific antigen levels |
| Pourreza et al. Iran 2021 (66) |  |  |  |  |  |  | x |  | Sleep quality and inflammatory markers |
| Ratjen et al. Germany 2020 (67) |  |  |  |  |  |  |  | x | Measures of abdominal fat distribution and liver fat content. |
| Ratjen et al. Germany 2021 (68) |  |  |  |  |  |  |  | x | All-cause mortality |
| Salomé et al. France 2021 (70) |  |  |  |  |  |  |  | x | Food processing, patterns of protein intake, and cardiometabolic risk. |
| Satija et al. US 2019 (71) |  |  |  |  |  |  |  | x | Weight change |
| Shirzadi et al. Iran 2022 (74) |  | x |  |  |  | x | x |  | CVD risk factors |
| Stanford et al. Australia 2021 (75) |  | x |  |  |  |  | x |  | Uremic Toxins, and Gut Microbiota |
| Wang et al. Australia 2021 (76) |  |  |  |  |  | x | x |  | Risk of obesity |
| Waterplas et al. Belgium 2020 (77) |  |  |  |  |  |  |  | x | Anthropometric parameters and blood lipids |
| Weber et al. Germany 2022 (78) |  |  |  |  |  |  |  | x | Plasma boron concentrations |
| Weston et al. US 2022 (79) | x |  |  | x |  |  |  |  | Incident cardiovascular disease and all-cause mortality |
| Wu et al. Singapore 2019 (80) |  |  | x |  |  |  | x |  | Cognitive impairment in later life |
| Yue et al. US 2021 (81) |  |  |  |  |  |  |  | x | Risk of colorectal cancer |
| Zamani et al. Iran 2020 (83) |  |  |  |  |  | x | x |  | Psychological profile and obesity |
| Zhou et al. Singapore 2021 (84) |  |  | x |  |  |  | x |  | Healthy ageing |
| **Plant-based dietary index (n=2)** (34) | | | | | | | | | |
| Chen et al., Netherlands 2019 (109) |  |  |  |  |  |  |  | x | Adiposity |
| Kim et al. US 2020 (49) |  | x |  |  |  |  | x |  | Risk of incident hypertension |
| **Plant-based Diet Quality Index (pDQI) (n=1)** (2) | | | | | | | | | |
| Brunin et al. France 2022 (3) |  |  |  |  |  |  |  | x | Dietary patterns, organic food consumption and socio-demographics |
| **Prime Diet Quality Score (PDQS) (n=3)** (110) | | | | | | | | | |
| Alvarez-Alvarez et al., 2020 Spain (111) |  |  |  |  |  |  |  | x | Hypertension, obesity, diabetes, and dyslipidaemia |
| Gu et al. US 2022 (112) |  |  |  |  |  |  |  | x | Risk of overall heart failure |
| Yue et al. US 2021 (81) |  |  |  |  |  |  |  | x | Risk of colorectal cancer |
| **Provegetarian food pattern (n=18)** (113) | | | | | | | | | |
| Alvarez-Alvarez et al., 2020 Spain (111) |  |  |  |  |  |  |  | x | Hypertension, obesity, diabetes, and dyslipidaemia |
| Fresán et al. Spain 2019 (114) |  |  |  |  |  |  |  | x | Sustainability of the dietary patterns |
| Gómez-Donozo et al. Spain 2019 (115) |  |  |  |  |  |  |  | x | Overweight/obesity incidence |
| Kane-Diallo et al. France 2018 (116) |  | x |  |  |  |  |  |  | Cancer risk |
| Kim et al. South Korea 2020 (46) |  |  | x |  |  |  | x |  | Incident metabolic syndrome |
| Kim et al. US 2019 (48) |  |  | x |  |  |  | x |  | Incident cardiovascular disease, cardiovascular disease mortality, and all-cause mortality |
| Kim et al. US 2020 (49) |  |  | x |  |  |  | x |  | Risk of incident hypertension |
| Leone et al. Spain 2020 (117) |  |  |  |  |  |  |  | x | Risk of basal cell carcinoma |
| Mohamadi et al. Iran 2022 (61) |  | x |  |  |  | x | x |  | Metabolically healthy obesity and metabolically unhealthy obesity phenotypes |
| Munoz-Garcia et al. Spain 2020 (118) |  |  |  |  |  |  |  | x | Cognitive function |
| Oncina‐Cánovas et al. Spain 2022 (65) |  |  |  |  |  |  |  | x | Cardiometabolic risk |
| Pano et al. Spain 2020 (119) |  |  |  |  |  |  |  | x | Health related quality of life |
| Pimenta et al. Spain 2015 (120) |  |  |  |  |  |  |  | x | Metabolic syndrome |
| Romanos-Nanclares et al. Spain 2020 (69) | x |  |  |  |  |  | x |  | Breast Cancer incidence |
| Salas-Huetos et al. US 2022 (121) |  |  |  |  |  | x |  |  | Semen parameters and couples’ assisted reproductive technology outcomes |
| Sánchez-Villegas et al. Spain 2015 (122) |  |  |  |  |  |  |  | x | Depression |
| Shahavandi et al. Iran 2020 (72) |  | x |  |  |  |  | x |  | Visceral adiposity, lipid accumulation product, and triglyceride-glucose index |
| Zaspe et al. Spain 2021 (123) |  |  |  |  |  |  |  | x | Within-participant longitudinal changes in diet scores |
| **TOT-Diet score (n=1)** (124) | | | | | | | | | |
| van der Velde et al. Netherlands 2022 (125) |  |  |  |  |  |  |  | x | Food insecurity and fast food outlet exposure |
| **Unhealthful plant-based diet index (uPDI) (n=49)** (23) | | | | | | | | | |
| Abaj et al. Iran 2022 (24) |  |  |  |  |  |  |  | x | Caveolin‐1, metabolic factors, serum inflammatory markers and anthropometric measures |
| Aljuraiban et al. Saudi Arabia 2022 (25) |  |  |  |  |  | x | x |  | High-sensitivity C-reactive protein level |
| Aljuraiban Saudi Arabia 2022 (26) |  |  |  |  |  | x | x |  | Psychological stress |
| Amini et al. Iran 2021 (27) |  |  |  |  |  | x | x |  | Metabolic syndrome |
| Anyene et al. US 2021 (28) |  |  |  |  |  |  |  | x | Breast Cancer Recurrence and Survival |
| Baden et al. US 2019 (29) |  |  |  |  |  |  |  | x | Total and cause-specific mortality |
| Baden et al. US 2019 (30) |  |  |  |  |  |  |  | x | Plasma adiposity-associated biomarker concentrations |
| Baden et al. US 2020 (31) |  |  |  |  |  |  |  | x | Health-related quality of life |
| Bolori et al. Iran 2019 (32) |  |  |  |  |  |  |  | x | Levels of liver enzymes, inflammatory factors and adipocytes profile |
| Brunin et al. France 2022 (3) |  |  |  |  |  |  |  | x | Dietary patterns, organic food consumption and socio-demographics |
| Chen et al., US 2021 (35) |  |  |  |  |  |  |  | x | Risk of type 2 diabetes |
| Daneshzad et al. Iran 2020 (37) |  |  |  |  |  | x | x |  | Sleep status and mental health |
| Flores et al. US 2021 (38) |  |  |  |  |  |  |  | x | Risk of developing diabetes |
| Gehring et al. France 2020 (39) |  |  |  |  |  |  |  | x | Consumption of UPFs |
| Gómez-Donozo et al. Spain 2019 (40) |  |  |  |  |  |  |  | x | Overweight/obesity incidence |
| Grisotto et al. US 2022 (42) |  |  |  |  |  |  |  | x | Early onset of natural menopause |
| Kawasaki et al. Japan 2021 (45) | x |  |  |  |  | x | x |  | Chronotype |
| Kim et al. South Korea 2020 (46) | x |  | x |  |  |  | x |  | Incident metabolic syndrome |
| Kim et al. South Korea 2021 (47) | x |  | x |  |  |  | x |  | Risk of hypertension |
| Kim et al. US 2019 (48) |  |  | x |  |  |  | x |  | Incident cardiovascular disease, cardiovascular disease mortality, and all-cause mortality |
| Kim et al. US 2020 (49) |  |  | x |  |  |  | x |  | Risk of incident hypertension |
| Kim et al. South Korea 2021 (50) | x |  | x | x |  |  | x |  | Risk of total and disease-specific mortality |
| Kim et al. South Korea 2022 (51) | x |  | x | x |  |  | x |  | Risk of type 2 diabetes |
| Kouvari et al. Greece 2022 (52) | x |  |  |  |  |  | x |  | Fatal/non-fatal cardiovascular disease incidence |
| Laouali et al. France 2021 (53) |  |  |  |  |  |  |  | x | Type 2 diabetes and hypertension risks |
| Lee et al. Korea 2021 (54) | x |  | x |  |  |  | x |  | Risk of dyslipidemia, and individual lipid disorders |
| Li et al. US 2022 (56) |  |  |  |  |  |  |  | **x** | Mortality risk |
| Liang et al. China 2022 (57) | x |  |  |  |  |  | x |  | Cognitive impairment |
| Lim et al. Singapore 2022 (58) |  |  | x |  |  | x |  |  | Fecundability |
| Loeb et al. US 2022 (59) |  |  |  |  |  |  |  | x | Prostate cancer risk |
| Mohamadi et al. Iran 2022 (61) |  |  |  |  |  | x | x |  | Metabolically healthy obesity and metabolically unhealthy obesity phenotypes |
| Mousavi et al. Iran 2021 (62) |  |  |  |  |  |  |  | x | Risk of glioma |
| Mousavi et al. Iran 2022 (63) |  |  |  |  |  |  |  | x | Psychological disorders |
| Oncina‐Cánovas et al. Spain 2022 (65) |  | x |  |  |  |  | x |  | Cardiometabolic risk |
| Pourreza et al. Iran 2021 (66) |  |  |  |  |  |  | x |  | Sleep quality and inflammatory markers |
| Ratjen et al. Germany 2020 (67) |  |  |  |  |  |  |  | x | Measures of abdominal fat distribution and liver fat content. |
| Ratjen et al. Germany 2021 (68) |  |  |  |  |  |  |  | x | All-cause mortality |
| Romanos-Nanclares et al. Spain 2020 (69) | x |  |  |  |  |  | x |  | Breast Cancer incidence |
| Salomé et al. France 2021 (70) |  |  |  |  |  |  |  | x | Food processing, patterns of protein intake, and cardiometabolic risk. |
| Satija et al. US 2019 (71) |  |  |  |  |  |  |  | x | Weight change |
| Shahavandi et al. Iran 2020 (72) |  |  |  |  |  |  |  | x | Visceral adiposity, lipid accumulation product, and triglyceride-glucose index |
| Shirzadi et al. Iran 2022 (74) |  | x |  |  |  | x | x |  | CVD risk factors |
| Stanford et al. Australia 2021 (75) |  | x |  |  |  |  | x |  | Uremic Toxins, and Gut Microbiota |
| Wang et al. Australia 2021 (76) |  |  |  |  |  | x | x |  | Risk of obesity |
| Waterplas et al. Belgium 2020 (77) |  |  |  |  |  |  |  | x | Anthropometric parameters and blood lipids |
| Weber et al. Germany 2022 (78) |  |  |  |  |  |  |  | x | Plasma boron concentrations |
| Weston et al. US 2022 (79) | x |  |  | x |  |  |  |  | Incident cardiovascular disease and all-cause mortality |
| Yue et al. US 2021 (81) |  |  |  |  |  |  |  | x | Risk of colorectal cancer |
| Zamani et al. Iran 2020 (83) |  |  |  |  |  | x | x |  | Psychological profile and obesity |
| **Totals** | **n=33 15.2%** | **n=21 9.7%** | **n=41 18.9%** | **n=15 6.9%** | **n=3 1.4%** | **n=35 16.1%** | **n=91 41.9%** | **n=103 47.5%** |  |

**References:**

1. Tricco AC, Lillie E, Zarin W, O'Brien KK, Colquhoun H, Levac D, et al. PRISMA Extension for Scoping Reviews (PRISMA-ScR): Checklist and Explanation. Ann Intern Med. 2018;169(7):467-73.

2. Keaver L, Ruan M, Chen F, Du M, Ding C, Wang J, et al. Plant- and animal-based diet quality and mortality among US adults: a cohort study. British Journal of Nutrition. 2021;125(12):1405-15.

3. Brunin J, Pointereau P, Allès B, Touvier M, Hercberg S, Lairon D, et al. Are recent dietary changes observed in the NutriNet-Santé participants healthier and more sustainable? European Journal of Nutrition. 2022;61(1):141-55.

4. Meyer KA, Sijtsma FPC, Nettleton JA, Steffen LM, Van Horn L, Shikany JM, et al. Dietary patterns are associated with plasma F₂-isoprostanes in an observational cohort study of adults. Free radical biology & medicine. 2013;57:201-9.

5. Gray ME, Bae S, Ramachandran R, Baldwin N, VanWagner LB, Jacobs Jr DR, et al. Dietary Patterns and Prevalent NAFLD at Year 25 from the Coronary Artery Risk Development in Young Adults (CARDIA) Study. Nutrients. 2022;14(4):854.

6. Vincent HK, Bourguignon CM, Taylor AG. Relationship of the dietary phytochemical index to weight gain, oxidative stress and inflammation in overweight young adults. Journal of human nutrition and dietetics : the official journal of the British Dietetic Association. 2010;23(1):20-9.

7. Amirkhizi F, Ghoreishy SM, Hamedi-Shahraki S, Asghari S. Higher dietary phytochemical index is associated with lower odds of knee osteoarthritis. Scientific Reports. 2022;12(5).

8. Asgari E, Jayedi A, Firouzabadi FD, Noruzi Z, Farazi M, Djafarian K, et al. Association of the dietary phytochemical index with general and central obesity in a sample of Iranian adults. Journal of Functional Foods. 2021;83.

9. Bahadoran Z, Golzarand M, Mirmiran P, Saadati N, Azizi F. The association of dietary phytochemical index and cardiometabolic risk factors in adults: Tehran Lipid and Glucose Study. Journal of human nutrition and dietetics : the official journal of the British Dietetic Association. 2013;26 Suppl 1:145-53.

10. Esmaeily Z, Sotoudeh G, Rafiee M, Koohdani F. ApoA2–256T > C polymorphism interacts with Healthy Eating Index, Dietary Quality Index-International and Dietary Phytochemical Index to affect biochemical markers among type 2 diabetic patients. British Journal of Nutrition. 2022;127(9):1343-51.

11. Ghoreishy SM, Aminianfar A, Benisi-Kohansal S, Azadbakht L, Esmaillzadeh A. Association between dietary phytochemical index and breast cancer: a case-control study. Breast cancer (Tokyo, Japan). 2021;28(6):1283-91.

12. Mirzababaei A, Taheri A, Rasaei N, Mehranfar S, Jamili S, Clark CCT, et al. The relationship between dietary phytochemical index and resting metabolic rate mediated by inflammatory factors in overweight and obese women: a cross-sectional study. BMC women's health. 2022;22(1):313.

13. Noruzi Z, Shiraseb F, Mirzababaei A, Mirzaei K. Association of the dietary phytochemical index with circadian rhythm and mental health in overweight and obese women: a cross-sectional study. Clinical Nutrition ESPEN. 2022;48:393-400.

14. Caivano S, Colugnati FAB, Domene SMA. Diet Quality Index associated with Digital Food Guide: update and validation. Cad Saude Publica. 2019;35(9):e00043419.

15. Assumpção Dd, Caivano S, Corona LP, Barros MBdA, Barros Filho AdA, Domene SMÁ. Diet quality among older adults: What the Index Associated with the Digital Food Guide and the Brazilian Healthy Eating Index-Revised Reveal. Ciencia & saude coletiva. 2022;27(4):1477-90.

16. van der Velde LA, van Dijk WW, Numans ME, Kiefte-de Jong JC. Extending the Theory of Planned Behavior for Explaining Dietary Quality: The Role of Financial Scarcity and Food Insecurity Status. Journal of nutrition education and behavior. 2022;54(7):636-46.

17. Bhattacharya R, Zekavat SM, Uddin MM, Pirruccello J, Niroula A, Gibson C, et al. Association of Diet Quality With Prevalence of Clonal Hematopoiesis and Adverse Cardiovascular Events. JAMA cardiology. 2021;6(9):1069-77.

18. Bromage S, Batis C, Bhupathiraju SN, Fawzi WW, Fung TT, Li Y, et al. Development and Validation of a Novel Food-Based Global Diet Quality Score (GDQS). J Nutr. 2021;151(12 Suppl 2):75S-92S.

19. Angulo E, Stern D, Castellanos-Gutiérrez A, Monge A, Lajous M, Bromage S, et al. Changes in the Global Diet Quality Score, Weight, and Waist Circumference in Mexican Women. The Journal of nutrition. 2021;151(12 Suppl 2):152S-61S.

20. Birk N, Matsuzaki M, Fung TT, Li Y, Batis C, Stampfer MJ, et al. Exploration of Machine Learning and Statistical Techniques in Development of a Low-Cost Screening Method Featuring the Global Diet Quality Score for Detecting Prediabetes in Rural India. The Journal of nutrition. 2021;151(12 Suppl 2):110S-8S.

21. Fung TT, Li Y, Bhupathiraju SN, Bromage S, Batis C, Holmes MD, et al. Higher Global Diet Quality Score Is Inversely Associated with Risk of Type 2 Diabetes in US Women. The Journal of nutrition. 2021;151(12 Suppl 2):168S-75S.

22. He Y, Fang Y, Bromage S, Fung TT, Bhupathiraju SN, Batis C, et al. Application of the Global Diet Quality Score in Chinese Adults to Evaluate the Double Burden of Nutrient Inadequacy and Metabolic Syndrome. The Journal of nutrition. 2021;151(12 Suppl 2):93S-100S.

23. Satija A, Bhupathiraju SN, Rimm EB, Spiegelman D, Chiuve SE, Borgi L, et al. Plant-Based Dietary Patterns and Incidence of Type 2 Diabetes in US Men and Women: Results from Three Prospective Cohort Studies. PLoS medicine. 2016;13(6):e1002039.

24. Abaj F, Mirzababaei A, Hosseininasab D, Bahrampour N, Clark CCT, Mirzaei K. Interactions between Caveolin-1 polymorphism and Plant-based dietary index on metabolic and inflammatory markers among women with obesity. Scientific reports. 2022;12(1):9088.

25. Aljuraiban GS, Gibson R, Leenah A-F, Sara A-M, Shivappa N, Hébert JR, et al. Associations among plant-based dietary indexes, the dietary inflammatory index, and inflammatory potential in female college students in Saudi Arabia: a cross-sectional study. Journal of the Academy of Nutrition and Dietetics. 2022;122(4):771-85.e8.

26. Aljuraiban GS. Plant-based dietary indices and stress in female college students: a cross-sectional study. British Journal of Nutrition. 2022;127(1):123-32.

27. Amini MR, Shahinfar H, Djafari F, Sheikhhossein F, Naghshi S, Djafarian K, et al. The association between plant-based diet indices and metabolic syndrome in Iranian older adults. Nutrition and health. 2021;27(4):435-44.

28. Anyene IC, Ergas IJ, Kwan ML, Roh JM, Ambrosone CB, Kushi LH, et al. Plant-Based Dietary Patterns and Breast Cancer Recurrence and Survival in the Pathways Study. Nutrients. 2021;13(10).

29. Baden MY, Liu G, Satija A, Li Y, Sun Q, Fung TT, et al. Changes in Plant-Based Diet Quality and Total and Cause-Specific Mortality. Circulation. 2019;140(12):979-91.

30. Baden MY, Satija A, Hu FB, Huang T. Change in Plant-Based Diet Quality Is Associated with Changes in Plasma Adiposity-Associated Biomarker Concentrations in Women. The Journal of nutrition. 2019;149(4):676-86.

31. Baden MY, Kino S, Liu X, Li Y, Kim Y, Kubzansky LD, et al. Changes in plant-based diet quality and health-related quality of life in women. The British journal of nutrition. 2020;124(9):960-70.

32. Bolori P, Setaysh L, Rasaei N, Jarrahi F, Yekaninejad MS, Mirzaei K. Adherence to a healthy plant diet may reduce inflammatory factors in obese and overweight women-a cross-sectional study. Diabetes & metabolic syndrome. 2019;13(4):2795-802.

33. Carto C, Pagalavan M, Nackeeran S, Blachman-Braun R, Kresch E, Kuchakulla M, et al. Consumption of a Healthy Plant-based Diet is Associated With a Decreased Risk of Erectile Dysfunction: A Cross-sectional Study of the National Health and Nutrition Examination Survey. Urology. 2022;161:76-82.

34. Chen Z, Zuurmond MG, van der Schaft N, Nano J, Wijnhoven HAH, Ikram MA, et al. Plant versus animal based diets and insulin resistance, prediabetes and type 2 diabetes: the Rotterdam Study. European journal of epidemiology. 2018;33(9):883-93.

35. Chen Z, Drouin-Chartier JP, Li Y, Baden MY, Manson JE, Willett WC, et al. Changes in plant-based diet indices and subsequent risk of type 2 diabetes in women and men: three U.S. prospective cohorts. Diabetes Care. 2021;44(3):663-71.

36. Chen G-C, Chai JC, Xing J, Moon J-Y, Shan Z, Yu B, et al. Healthful eating patterns, serum metabolite profile and risk of diabetes in a population-based prospective study of US Hispanics/Latinos. Diabetologia. 2022;65(7):1133-44.

37. Daneshzad E, Keshavarz S-A, Qorbani M, Larijani B, Bellissimo N, Azadbakht L. Association of dietary acid load and plant-based diet index with sleep, stress, anxiety and depression in diabetic women. The British journal of nutrition. 2020;123(8):901-12.

38. Flores AC, Heron C, Kim JI, Martin B, Al-Shaar L, Tucker KL, et al. Prospective Study of Plant-Based Dietary Patterns and Diabetes in Puerto Rican Adults. The Journal of nutrition. 2021;151(12):3795-800.

39. Gehring J, Touvier M, Baudry J, Julia C, Buscail C, Srour B, et al. Consumption of Ultra-Processed Foods by Pesco-Vegetarians, Vegetarians, and Vegans: Associations with Duration and Age at Diet Initiation. The Journal of nutrition. 2021;151(1):120-31.

40. Gómez-Donoso C, Martínez-González MA, Gea A, Murphy KJ, Parletta N, Bes-Rastrollo M. A food-based score and incidence of overweight/obesity: The Dietary Obesity-Prevention Score (DOS). Clinical nutrition (Edinburgh, Scotland). 2019;38(6):2607-15.

41. González-Ortiz A, Xu H, Ramos-Acevedo S, Avesani CM, Lindholm B, Correa-Rotter R, et al. Nutritional status, hyperkalaemia and attainment of energy/protein intake targets in haemodialysis patients following plant-based diets: a longitudinal cohort study. Nephrology, dialysis, transplantation : official publication of the European Dialysis and Transplant Association - European Renal Association. 2021;36(4):681-8.

42. Grisotto G, Langton CR, Li Y, Bertone-Johnson ER, Baden MY, Franco OH, et al. Association of plant-based diet and early onset of natural menopause. Menopause (New York, NY). 2022;29(7):861-7.

43. Heianza Y, Zhou T, Sun D, Hu FB, Manson JE, Qi L. Genetic susceptibility, plant-based dietary patterns, and risk of cardiovascular disease. The American journal of clinical nutrition. 2020;112(1):220-8.

44. Heianza Y, Zhou T, Sun D, Hu FB, Qi L. Healthful plant-based dietary patterns, genetic risk of obesity, and cardiovascular risk in the UK biobank study. Clinical Nutrition. 2021;40(7):4694-701.

45. Kawasaki Y, Akamatsu R, Fujiwara Y, Omori M, Sugawara M, Yamazaki Y, et al. Later chronotype is associated with unhealthful plant-based diet quality in young Japanese women. Appetite. 2021;166.

46. Kim H, Lee K, Rebholz CM, Kim J. Plant-based diets and incident metabolic syndrome: Results from a South Korean prospective cohort study. PLoS Medicine. 2020;17(11):e1003371.

47. Kim J, Kim H, Giovannucci EL. Quality of plant-based diets and risk of hypertension: a Korean genome and examination study. European Journal of Nutrition. 2021;60(7):3841-51.

48. Kim H, Caulfield LE, Garcia-Larsen V, Steffen LM, Coresh J, Rebholz CM. Plant-Based Diets Are Associated With a Lower Risk of Incident Cardiovascular Disease, Cardiovascular Disease Mortality, and All-Cause Mortality in a General Population of Middle-Aged Adults. Journal of the American Heart Association. 2019;8(16):e012865.

49. Kim H, Rebholz CM, Garcia-Larsen V, Steffen LM, Coresh J, Caulfield LE. Operational Differences in Plant-Based Diet Indices Affect the Ability to Detect Associations with Incident Hypertension in Middle-Aged US Adults. The Journal of nutrition. 2020;150(4):842-50.

50. Kim J, Kim H, Giovannucci EL. Plant-based diet quality and the risk of total and disease-specific mortality: A population-based prospective study. Clinical Nutrition. 2021;40(12):5718-25.

51. Kim J, Giovannucci E. Healthful Plant-Based Diet and Incidence of Type 2 Diabetes in Asian Population. Nutrients. 2022;14(15).

52. Kouvari M, Tsiampalis T, Chrysohoou C, Georgousopoulou E, Skoumas J, Mantzoros CS, et al. Quality of plant-based diets in relation to 10-year cardiovascular disease risk: the ATTICA cohort study. European Journal of Nutrition. 2022;61(5):2639-49.

53. Laouali N, Shah S, MacDonald CJ, Mahamat-Saleh Y, El-Fatouhi D, Mancini F, et al. BMI in the associations of plant-based diets with type 2 diabetes and hypertension risks in women: the E3N prospective cohort study. Journal of Nutrition. 2021;151(9):2731-40.

54. Lee K, Kim H, Rebholz CM, Kim J. Association between Different Types of Plant-Based Diets and Risk of Dyslipidemia: A Prospective Cohort Study. Nutrients. 2021;13(1).

55. Li Y, Wang DD, Satija A, Ivey KL, Li J, Wilkinson JE, et al. Plant-based diet index and metabolic risk in men: exploring the role of the gut microbiome. Journal of Nutrition. 2021;151(9):2780-9.

56. Li H, Zeng X, Wang Y, Zhang Z, Zhu Y, Li X, et al. A prospective study of healthful and unhealthful plant-based diet and risk of overall and cause-specific mortality. European journal of nutrition. 2022;61(1):387-98.

57. Liang F, Fu J, Turner-McGrievy G, Wang Y, Qiu N, Ding K, et al. Association of Body Mass Index and Plant-Based Diet with Cognitive Impairment among Older Chinese Adults: A Prospective, Nationwide Cohort Study. Nutrients. 2022;14(15).

58. Lim SX, Loy SL, Colega MT, Lai JS, Godfrey KM, Lee YS, et al. Prepregnancy adherence to plant-based diet indices and exploratory dietary patterns in relation to fecundability. The American journal of clinical nutrition. 2022;115(2):559-69.

59. Loeb S, Fu BC, Bauer SR, Pernar CH, Chan JM, Van Blarigan EL, et al. Association of plant-based diet index with prostate cancer risk. American Journal of Clinical Nutrition. 2022;115(3):662-70.

60. Merino J, Joshi AD, Nguyen LH, Leeming ER, Mazidi M, Drew DA, et al. Diet quality and risk and severity of COVID-19: a prospective cohort study. Gut. 2021;70(11):2096-104.

61. Mohamadi A, Shiraseb F, Mirzababaei A, Hosseininasab D, Rasaei N, Clark CCT, et al. Circulating Inflammatory Markers May Mediate the Relationship between Healthy Plant-Based Diet and Metabolic Phenotype Obesity in Women: A Cross-Sectional Study. International journal of clinical practice. 2022;2022:8099382.

62. Mousavi SM, Shayanfar M, Rigi S, Mohammad-Shirazi M, Sharifi G, Esmaillzadeh A. Adherence to plant-based dietary patterns in relation to glioma: a case-control study. Scientific reports. 2021;11(1):21819.

63. Mousavi SM, Ebrahimi-Mousavi S, Hassanzadeh Keshteli A, Afshar H, Esmaillzadeh A, Adibi P. The association of plant-based dietary patterns and psychological disorders among Iranian adults. Journal of affective disorders. 2022;300:314-21.

64. Mouzannar A, Kuchakulla M, Blachman-Braun R, Nackeeran S, Becerra M, Nahar B, et al. Impact of Plant-Based Diet on PSA Level: Data From the National Health and Nutrition Examination Survey. Urology. 2021;156:205-10.

65. Oncina-Cánovas A, Vioque J, González-Palacios S, Martínez-González MÁ, Salas-Salvadó J, Corella D, et al. Pro-vegetarian food patterns and cardiometabolic risk in the PREDIMED-Plus study: a cross-sectional baseline analysis. European Journal of Nutrition. 2022;61(1):357-72.

66. Pourreza S, Khademi Z, Mirzababaei A, Yekaninejad MS, Sadeghniiat-Haghighi K, Naghshi S, et al. Association of plant-based diet index with inflammatory markers and sleep quality in overweight and obese female adults: A cross-sectional study. International journal of clinical practice. 2021;75(9):e14429.

67. Ratjen I, Morze J, Enderle J, Both M, Borggrefe J, Müller H-P, et al. Adherence to a plant-based diet in relation to adipose tissue volumes and liver fat content. The American journal of clinical nutrition. 2020;112(2):354-63.

68. Ratjen I, Enderle J, Burmeister G, Koch M, Nöthlings U, Hampe J, et al. Post-diagnostic reliance on plant-compared with animal-based foods and all-cause mortality in omnivorous long-term colorectal cancer survivors. The American journal of clinical nutrition. 2021;114(2):441-9.

69. Romanos-Nanclares A, Toledo E, Sánchez-Bayona R, Sánchez-Quesada C, Martínez-González MÁ, Gea A. Healthful and unhealthful provegetarian food patterns and the incidence of breast cancer: results from a Mediterranean cohort. Nutrition. 2020;79/80.

70. Salomé M, Arrazat L, Wang J, Dufour A, Dubuisson C, Volatier J-L, et al. Contrary to ultra-processed foods, the consumption of unprocessed or minimally processed foods is associated with favorable patterns of protein intake, diet quality and lower cardiometabolic risk in French adults (INCA3). European Journal of Nutrition. 2021;60(7):4055-67.

71. Satija A, Malik V, Rimm EB, Sacks F, Willett W, Hu FB. Changes in intake of plant-based diets and weight change: results from 3 prospective cohort studies. The American journal of clinical nutrition. 2019;110(3):574-82.

72. Shahavandi M, Djafari F, Shahinfar H, Davarzani S, Babaei N, Ebaditabar M, et al. The association of plant-based dietary patterns with visceral adiposity, lipid accumulation product, and triglyceride-glucose index in Iranian adults. Complementary therapies in medicine. 2020;53:102531.

73. Shan Z, Li Y, Baden MY, Bhupathiraju SN, Wang DD, Sun Q, et al. Association Between Healthy Eating Patterns and Risk of Cardiovascular Disease. JAMA Internal Medicine. 2020;180(8):1090-100.

74. Shirzadi Z, Daneshzad E, Dorosty A, Surkan PJ, Azadbakht L. Associations of plant-based dietary patterns with cardiovascular risk factors in women. Journal of Cardiovascular & Thoracic Research. 2022;14(1):1-10.

75. Stanford J, Charlton K, Stefoska-Needham A, Zheng H, Bird L, Borst A, et al. Associations among plant-based diet quality, uremic toxins, and gut microbiota profile in adults undergoing hemodialysis therapy. Journal of Renal Nutrition. 2021;31(2):177-88.

76. Wang YB, Shivappa N, Hébert JR, Page AJ, Gill TK, Melaku YA. Association between Dietary Inflammatory Index, Dietary Patterns, Plant-Based Dietary Index and the Risk of Obesity. Nutrients. 2021;13(5).

77. Waterplas J, Versele V, D'Hondt E, Lefevre J, Mertens E, Charlier R, et al. A 10-year longitudinal study on the associations between changes in plant-based diet indices, anthropometric parameters and blood lipids in a Flemish adult population. Nutrition & dietetics: the journal of the Dietitians Association of Australia. 2020;77(2):196-203.

78. Weber KS, Ratjen I, Enderle J, Seidel U, Rimbach G, Lieb W. Plasma boron concentrations in the general population: a cross-sectional analysis of cardio-metabolic and dietary correlates. European journal of nutrition. 2022;61(3):1363-75.

79. Weston LJ, Kim H, Talegawkar SA, Tucker KL, Correa A, Rebholz CM. Plant-based diets and incident cardiovascular disease and all-cause mortality in African Americans: A cohort study. PLoS medicine. 2022;19(1):e1003863.

80. Wu J, Song X, Chen G-C, Neelakantan N, van Dam RM, Feng L, et al. Dietary pattern in midlife and cognitive impairment in late life: a prospective study in Chinese adults. The American journal of clinical nutrition. 2019;110(4):912-20.

81. Yue Y, Hur J, Cao Y, Tabung FK, Wang M, Wu K, et al. Prospective evaluation of dietary and lifestyle pattern indices with risk of colorectal cancer in a cohort of younger women. Annals of oncology : official journal of the European Society for Medical Oncology. 2021;32(6):778-86.

82. Zagarins SE, Ronnenberg AG, Bertone-Johnson ER. Established diet quality indices are not universally associated with body composition in young adult women. Public Health Nutrition. 2021;24(9):2465-72.

83. Zamani B, Daneshzad E, Siassi F, Guilani B, Bellissimo N, Azadbakht L. Association of plant-based dietary patterns with psychological profile and obesity in Iranian women. Clinical nutrition (Edinburgh, Scotland). 2020;39(6):1799-808.

84. Zhou Y-F, Song X-Y, Wu J, Chen G-C, Neelakantan N, van Dam RM, et al. Association Between Dietary Patterns in Midlife and Healthy Ageing in Chinese Adults: The Singapore Chinese Health Study. Journal of the American Medical Directors Association. 2021;22(6):1279-86.

85. Olsen A, Egeberg R, Halkjaer J, Christensen J, Overvad K, Tjonneland A. Healthy aspects of the Nordic diet are related to lower total mortality. J Nutr. 2011;141(4):639-44.

86. Gunge VB, Andersen I, Kyrø C, Hansen CP, Dahm CC, Christensen J, et al. Adherence to a healthy Nordic food index and risk of myocardial infarction in middle-aged Danes: the diet, cancer and health cohort study. European Journal of Clinical Nutrition. 2017;71(5):652-8.

87. Jensen E, M T, Braaten T, Jacobsen BK, Barnung RB, Skeie G, et al. Adherence to the Healthy Nordic Food Index in the Norwegian Women and Cancer (NOWAC) cohort. Food & Nutrition Research. 2018;62:1-11.

88. Li Y, Roswall N, Sandin S, Ström P, Adami H-O, Weiderpass E. Adherence to a healthy Nordic food index and breast cancer risk: results from a Swedish cohort study. Cancer causes & control : CCC. 2015;26(6):893-902.

89. Noruzi Z, Jayedi A, Farazi M, Moosavi H, Janbozorgi N, Djafarian K, et al. The Association Between the Nordic-Style Diet Score and Metabolic Syndrome and Obesity in Tehranian Adults. Nutrition Today. 2021;56(4):217-28.

90. Roswall N, Ängquist L, Ahluwalia TS, Romaguera D, Larsen SC, Østergaard JN, et al. Association between Mediterranean and Nordic diet scores and changes in weight and waist circumference: influence of FTO and TCF7L2 loci. The American journal of clinical nutrition. 2014;100(4):1188-97.

91. Roswall N, Eriksson U, Sandin S, Löf M, Olsen A, Skeie G, et al. Adherence to the healthy Nordic food index, dietary composition, and lifestyle among Swedish women. Food & Nutrition Research. 2015;59:1-10.

92. Tomata Y, Watanabe T, Sugawara Y, Chou W-T, Kakizaki M, Tsuji I. Dietary patterns and incident functional disability in elderly Japanese: the Ohsaki Cohort 2006 study. The journals of gerontology Series A, Biological sciences and medical sciences. 2014;69(7):843-51.

93. Lu Y, Matsuyama S, Sugawara Y, Sone T, Tsuji I. Changes in a specific dietary pattern and incident dementia: A prospective cohort study. Clinical Nutrition. 2021;40(5):3495-502.

94. Oba S, Nagata C, Nakamura K, Fujii K, Kawachi T, Takatsuka N, et al. Diet based on the Japanese Food Guide Spinning Top and subsequent mortality among men and women in a general Japanese population. J Am Diet Assoc. 2009;109(9):1540-7.

95. Kurotani K, Ishikawa-Takata K, Takimoto H. Diet quality of Japanese adults with respect to age, sex, and income level in the National Health and Nutrition Survey, Japan. Public Health Nutrition. 2020;23(5):821-32.

96. Watanabe D, Kurotani K, Yoshida T, Nanri H, Watanabe Y, Date H, et al. Diet quality and physical or comprehensive frailty among older adults. European journal of nutrition. 2022;61(5):2451-62.

97. Vinke PC, Corpeleijn E, Dekker LH, Jacobs DR, Jr., Navis G, Kromhout D. Development of the food-based Lifelines Diet Score (LLDS) and its application in 129,369 Lifelines participants. European journal of clinical nutrition. 2018;72(8):1111-9.

98. Cai Q, Dekker LH, Vinke PC, Corpeleijn E, Bakker SJL, de Borst MH, et al. Diet quality and incident chronic kidney disease in the general population: The Lifelines Cohort Study. Clinical nutrition (Edinburgh, Scotland). 2021;40(9):5099-105.

99. Moazzen S, Cortes-Ibañez FO, van der Vegt B, Alizadeh BZ, de Bock GH. Diet quality indices and gastrointestinal cancer risk: results from the Lifelines study. European Journal of Nutrition. 2022;61(1):317-27.

100. Sohouli MH, Hadizadeh M, Omrani M, Baniasadi M, Sanati V, Zarrati M. Adherence to Lifelines Diet Score (LLDS) Is Associated with a Reduced Risk of Breast Cancer (BrCa): A Case-Control Study. International journal of clinical practice. 2022;2022:7726126.

101. Galbete C, Kroger J, Jannasch F, Iqbal K, Schwingshackl L, Schwedhelm C, et al. Nordic diet, Mediterranean diet, and the risk of chronic diseases: the EPIC-Potsdam study. BMC Med. 2018;16(1):99.

102. Bonaccio M, Di Castelnuovo A, Costanzo S, De Curtis A, Persichillo M, Cerletti C, et al. Association of a traditional Mediterranean diet and non-Mediterranean dietary scores with all-cause and cause-specific mortality: prospective findings from the Moli-sani Study. European Journal of Nutrition. 2021;60(2):729-46.

103. Esposito S, Gialluisi A, Costanzo S, Di Castelnuovo A, Ruggiero E, De Curtis A, et al. Mediterranean diet and other dietary patterns in association with biological aging in the Moli-sani Study cohort. Clinical nutrition (Edinburgh, Scotland). 2022;41(5):1025-33.

104. Grosso G, Fresán U, Bes-Rastrollo M, Marventano S, Galvano F. Environmental Impact of Dietary Choices: Role of the Mediterranean and Other Dietary Patterns in an Italian Cohort. International Journal of Environmental Research and Public Health. 2020;17(5).

105. Liu D, Zhang X-R, Li Z-H, Zhang Y-J, Lv Y-B, Wang Z-H, et al. Association of dietary diversity changes and mortality among older people: A prospective cohort study. Clinical nutrition (Edinburgh, Scotland). 2021;40(5):2620-9.

106. Liu D, Zhang W-T, Wang J-H, Shen D, Zhang P-D, Li Z-H, et al. Association between Dietary Diversity Changes and Cognitive Impairment among Older People: Findings from a Nationwide Cohort Study. Nutrients. 2022;14(6).

107. González-Ortiz A, Xu H, Avesani CM, Lindholm B, Cederholm T, Risérus U, et al. Plant-based diets, insulin sensitivity and inflammation in elderly men with chronic kidney disease. Journal of nephrology. 2020;33(5):1091-101.

108. Lazarova SV, Sutherland JM, Jessri M. Adherence to emerging plant-based dietary patterns and its association with cardiovascular disease risk in a nationally representative sample of Canadian adults. The American journal of clinical nutrition. 2022;116(1):57-73.

109. Chen Z, Schoufour JD, Rivadeneira F, Lamballais S, Ikram MA, Franco OH, et al. Plant-based Diet and Adiposity Over Time in a Middle-aged and Elderly Population: The Rotterdam Study. Epidemiology (Cambridge, Mass). 2019;30(2):303-10.

110. Fung TT, Isanaka S, Hu FB, Willett WC. International food group-based diet quality and risk of coronary heart disease in men and women. The American journal of clinical nutrition. 2018;107(1):120-9.

111. Alvarez-Alvarez I, Toledo E, Lecea O, Salas-Salvadó J, Corella D, Buil-Cosiales P, et al. Adherence to a priori dietary indexes and baseline prevalence of cardiovascular risk factors in the PREDIMED-Plus randomised trial. European Journal of Nutrition. 2020;59(3):1219-32.

112. Gu X, Wang DD, Fung TT, Mozaffarian D, Djoussé L, Rosner B, et al. Dietary quality and risk of heart failure in men. American Journal of Clinical Nutrition. 2022;116(2):378-85.

113. Martínez-González MA, Sánchez-Tainta A, Corella D, Salas-Salvadó J, Ros E, Arós F, et al. A provegetarian food pattern and reduction in total mortality in the Prevención con Dieta Mediterránea (PREDIMED) study. American Journal of Clinical Nutrition. 2014;100:320S-8S.

114. Fresán U, Martínez-González MA, Sabaté J, Bes-Rastrollo M. Global sustainability (health, environment and monetary costs) of three dietary patterns: results from a Spanish cohort (the SUN project). BMJ Open. 2019;9(2):e021541.

115. Gómez-Donoso C, Martínez-González MÁ, Martínez JA, Gea A, Sanz-Serrano J, Perez-Cueto FJA, et al. A Provegetarian Food Pattern Emphasizing Preference for Healthy Plant-Derived Foods Reduces the Risk of Overweight/Obesity in the SUN Cohort. Nutrients. 2019;11(7).

116. Kane-Diallo A, Srour B, Sellem L, Deschasaux M, Latino-Martel P, Hercberg S, et al. Association between a pro plant-based dietary score and cancer risk in the prospective NutriNet-santé cohort. International Journal of Cancer. 2018;143(9):2168-76.

117. Leone A, Martínez-González MÁ, Martin-Gorgojo A, Sánchez-Bayona R, De Amicis R, Bertoli S, et al. Mediterranean diet, Dietary Approaches to Stop Hypertension, and Pro-vegetarian dietary pattern in relation to the risk of basal cell carcinoma: a nested case-control study within the Seguimiento Universidad de Navarra (SUN) cohort. The American journal of clinical nutrition. 2020;112(2):364-72.

118. Munoz-Garcia MI, Toledo E, Razquin C, Dominguez LJ, Maragarone D, Martinez-Gonzalez J, et al. "A priori" Dietary Patterns and Cognitive Function in the SUN Project. Neuroepidemiology. 2020;54(1):45-57.

119. Pano O, Sayón-Orea C, Gea A, Bes-Rastrollo M, Martínez-González MÁ, Martínez JA. Nutritional Determinants of Quality of Life in a Mediterranean Cohort: The SUN Study. International Journal of Environmental Research and Public Health. 2020;17(11).

120. Pimenta AM, Toledo E, Rodriguez-Diez MC, Gea A, Lopez-Iracheta R, Shivappa N, et al. Dietary indexes, food patterns and incidence of metabolic syndrome in a Mediterranean cohort: The SUN project. Clinical nutrition (Edinburgh, Scotland). 2015;34(3):508-14.

121. Salas-Huetos A, Mínguez-Alarcón L, Mitsunami M, Arvizu M, Ford JB, Souter I, et al. Paternal adherence to healthy dietary patterns in relation to sperm parameters and outcomes of assisted reproductive technologies. Fertility and sterility. 2022;117(2):298-312.

122. Sánchez-Villegas A, Henríquez-Sánchez P, Ruiz-Canela M, Lahortiga F, Molero P, Toledo E, et al. A longitudinal analysis of diet quality scores and the risk of incident depression in the SUN Project. BMC medicine. 2015;13:197.

123. Zazpe PI, Santiago PS, Toledo MDE, Bes-Rastrollo PM, Fuente-Arrillaga RDCdl, Martínez-González MDMÁ. Diet Quality Indices in the SUN Cohort: Observed Changes and Predictors of Changes in Scores Over a 10-Year Period. Journal of the Academy of Nutrition & Dietetics. 2021;121(10):1948-.

124. van der Velde LA, Nyns CJ, Engel MD, Neter JE, van der Meer IM, Numans ME, et al. Exploring food insecurity and obesity in Dutch disadvantaged neighborhoods: a cross-sectional mediation analysis. BMC Public Health. 2020;20(1):569.

125. van der Velde LA, Zitman FM, Mackenbach JD, Numans ME, Kiefte-de Jong JC. The interplay between fast-food outlet exposure, household food insecurity and diet quality in disadvantaged districts. Public health nutrition. 2022;25(1):105-13.
